# Diversity, Abundance and Community Composition of Birds in Chitwan Annapurna Landscape, Central Nepal

**DOI:** 10.1002/ece3.71781

**Published:** 2025-07-14

**Authors:** Shubhas Chandra Bastola, Pradip Kandel, Hathan Ram Mahato, Jagan Nath Adhikari, Bishnu Prasad Bhattarai

**Affiliations:** ^1^ Central Department of Zoology, Institute of Science and Technology Tribhuvan University Kathmandu Bagmati Nepal; ^2^ Department of Zoology Prithivi Narayan Campus Pokhara Kaski Nepal; ^3^ Himalayan Environment and Public Health Network (HEPHN) Bharatpur Chitwan Nepal; ^4^ Nepal Ornithological Union (NOU) Kathmandu Bagmati Nepal; ^5^ Department of Zoology Birendra Multiple Campus Chitwan Bagmati Nepal

**Keywords:** Annapurna Conservation Area, Chitwan National Park, habitats, migratory birds, threatened birds, wetlands

## Abstract

Understanding bird diversity and composition is important for assessing survival, adaptability, and extinction risks. This study investigated bird species abundance, richness, seasonal diversity, and composition across habitats, seasons, and physiographic zones of Chitwan Annapurna Landscape (CHAL) from 2020 to 2021 using the point count method. We recorded 458 bird species with higher richness and diversity in winter (*n* = 365) than in summer (*n* = 299). This study indicates a high diversity of birds (Shannon = 5.54, Simpson = 0.99) and an even distribution of species Pielou's Evenness Index (0.90). Species richness and abundance showed seasonal and habitat‐specific variations, with wetlands highest in winter (richness: 6.94 ± 0.38, abundance: 23.10 ± 5.65) and croplands in summer (richness: 7.00 ± 0.35, abundance: 13.61 ± 1.10), while forests exhibited stability. Evenness was high across all habitats (0.90 ± 0.01 to 0.95 ± 0.00), with minimal seasonal variation. Diversity indices were highest in cropland during summer (Margalef: 2.33 ± 0.10, Shannon: 1.77 ± 0.06, Simpson: 0.80 ± 0.02) and wetlands during winter (Margalef: 2.19 ± 0.08, Shannon: 1.75 ± 0.05, Simpson: 0.79 ± 0.01); forests had moderate and stable diversity, while grasslands showed low values. We observed 2 globally vulnerable, 5 near‐threatened, 2 endangered, and 3 critically endangered species. Analysis of similarity (ANOSIM) revealed significant community differentiation (*p* < 0.01) across parameters, with the strongest segregation in physiographic (*R* = 0.09), followed by seasons (*R* = 0.06) and habitat types (*R* = 0.01). Canonical Correspondence Analysis (CCA) showed distinct habitat clusters and a strong influence of physiographic zones, with winter and wetland habitats driving species variance. This study revealed substantial differences in bird species across seasons, and each habitat offers a distinct ecological niche for bird species. This finding highlights CHAL as a hotspot for bird community. This research contributes to the conservation and management of this landscape, aiding in the protection of bird species in Nepal.

## Graphical abstract

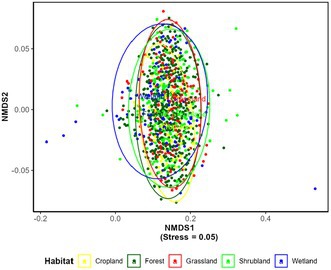

## 1 Introduction

Understanding the diversity and distribution of birds and other organisms is key to assessing their survival, adaptability, and extinction risks, enabling the protection of specific bird species and related biodiversity (Kremen 1992). Environmental factors such as climate, food availability, habitat fragmentation, and human activities shape bird population dynamics across different landscapes (Neuschulz et al. 2013). Forests support high biodiversity and provide stable environments for bird species with specialized diets and nesting behaviors, such as hornbills and woodpeckers (Baral and Huettmann 2020). Wetlands sustain both resident and migratory waterbirds like ducks and herons but are highly vulnerable to pollution and habitat loss (Szabo and Mundkur 2017). Grasslands and shrublands, home to ground‐nesting birds like bustards and larks, face threats from agriculture and grazing (Vickery et al. 2000; Ceballos et al. 2010). Urban areas offer abundant food sources for adaptable species like pigeons and crows but often limit the survival of habitat specialists, including many raptors (Adams 2018; Menon 2025). Bird survival, adaptability, and extinction risks vary across physiographic zones due to differences in habitat, climate, resources, and human activities (Fjeldså et al. 2012). Lowlands offer abundant food and water, but agriculture, urbanization, wetland drainage, pollution, and climate‐induced droughts threaten bird populations (Adhikari, Bhattarai, et al. 2022; Adhikari, Khatiwada, et al. 2022). River valleys, rich in food and nesting sites, serve as crucial habitats for many species. Mid‐hills and forested regions, though dominated by human activity, support diverse birdlife due to varied vegetation and altitude (Chettri et al. 2010). However, illegal logging, habitat destruction, and hunting pose significant threats. In contrast, mountain and alpine regions, with their harsh climate and scarce food, are home to specialized species like snowcocks and vultures. Yet, climate change and habitat shrinkage place these birds at high risk of extinction (Chettri et al. 2010). As altitude increases, the resources available to birds change due to differences in habitat structure, site productivity, forest types, vegetation composition, and land use patterns (Neupane, Khanal, et al. 2020; Neupane, Gyawali, et al. 2020). Variations in bird species diversity across elevation gradients are often noticed as an important aspect of community structure of the birds (Stevens 1992). This is because elevation influences the physical state of the surroundings that directly influences the breeding and foraging activities of the birds (Neupane, Khanal, et al. 2020; Neupane, Gyawali, et al. 2020). Seasonal shifts in bird communities are influenced by migration, breeding cycles, and changes in the availability of seasonal resources. These factors affect species composition, diversity, and abundance (Almazán‐Núnez et al. 2015; Katuwal et al. 2016; Pandey et al. 2020). Similarly, the diversity of birds is influenced by field margins, habitat types and quality, forest edges, habitat fragmentation, landscape changes, farming practices, vegetation types, and climate (Adhikari, Bhattarai, et al. 2022; Adhikari, Khatiwada, et al. 2022). Hence, understanding the composition of birds with their habitats is crucial for examining how biotic interactions influence the distribution of birds (Jankowski et al. 2013; Wiens 1989). Vegetation forms the backbone of terrestrial habitats for birds, offering shelter, food, foraging grounds, and breeding sites (Lee and Rotenberry 2005; Robinson and Holmes 1984). Birds are important bio‐indicators that help ecosystems function by pollinating plants, dispersing seeds, controlling harmful insects, recycling nutrients, and cleaning the environment by scavenging (Kiros et al. 2018).

Nepal harbors 897 species of birds (DNPWC and BCN 2022; Kathmandu Post 2024). About one‐third of the total bird species reported in Nepal are migratory. Around 62 species, including cuckoos, swifts, bee‐eaters, Phylloscopus warblers, flycatchers, and drongos, visit Nepal in the summer as seasonal or partial migrants (Inskipp et al. 2016a, 2016b). Around 150 species migrate to Nepal for the winter from across the Palearctic region. These include ducks, geese, birds of prey, waders, pipits, wagtails, thrushes, Acrocephalus, Locustella, and Phylloscopus warblers, as well as bush warblers, finches, and buntings (Inskipp et al. 2016a, 2016b). Among the reported species, 83 are vagrants and 29 are passage migrants (Inskipp et al. 2017). Besides long‐distance migrants, there are some birds that migrate across various altitudes, which move up and down the altitudinal gradients of the Himalayas, usually in response to seasonal changes in temperature, food availability, and breeding environments. As altitudinal variation and different habitat compositions of Nepal support 9.5% of the total birds species of the globe. Of these, 40 species are globally threatened, including 10 Critically Endangered, 7 Endangered, and 23 Vulnerable species, as per the IUCN Red List of Threatened Species (IUCN 2024). Similarly, the National Red Data Book identifies 168 nationally threatened species, comprising 68 Critically Endangered, 38 Endangered, and 62 Vulnerable species (Inskipp et al. 2016a, 2016b). Most of the studies on birds in Nepal are focused on the status, distribution, and diversity of a specific areas in different regions (Basnet et al. 2016; Katuwal et al. 2016; Adhikari et al. 2019; Khatri et al. 2019; Pandey et al. 2020; Ghimire et al. 2021; Bastola et al. 2022; Joshi et al. 2022; Lama et al. 2022; Neupane et al. 2022; Kunwar et al. 2023). Hence, research on the status, distribution, and diversity of the birds along with elevation gradients in Chitwan Annapurna Landscape (CHAL) is still scarce.

CHAL connects lowland Terai landscapes with the high mountain landscape. Bird survey in such areas is essential to update the biodiversity profile of Nepal and also support conservation efforts. In the CHAL, some of the research has been seen, but these are only concentrated in the small patches or protected areas (Giri and Chalise 2008; Khatri et al. 2019; Neupane, Khanal, et al. 2020; Neupane, Gyawali, et al. 2020; Rai et al. 2020; Basaula et al. 2021, 2023; Bastola 2021; Ghimire et al. 2021; Bastola et al. 2022; Poudel and Baral 2023; Thakuri et al. 2024). But species diversity and composition of the birds along with elevation gradients and habitat gradients in CHAL are still lacking. Our research aims to bridge knowledge gaps by exploring the current status, distribution, diversity, and composition of bird populations in the CHAL. This study contributes a complete and up‐toto‐date checklist of bird species present in the CHAL, filling gaps left by previous, localized research. This study analyzes the diversity and composition of bird communities across different seasons and habitats, revealing patterns and trends. Similarly, it identifies critical habitats for bird species in the CHAL, highlighting areas of high biodiversity and conservation importance, and also provides essential baseline data for developing and implementing effective conservation strategies for birds in the CHAL.

## 2 Materials and Methods

### 2.1 Study Area

**FIGURE 1:**
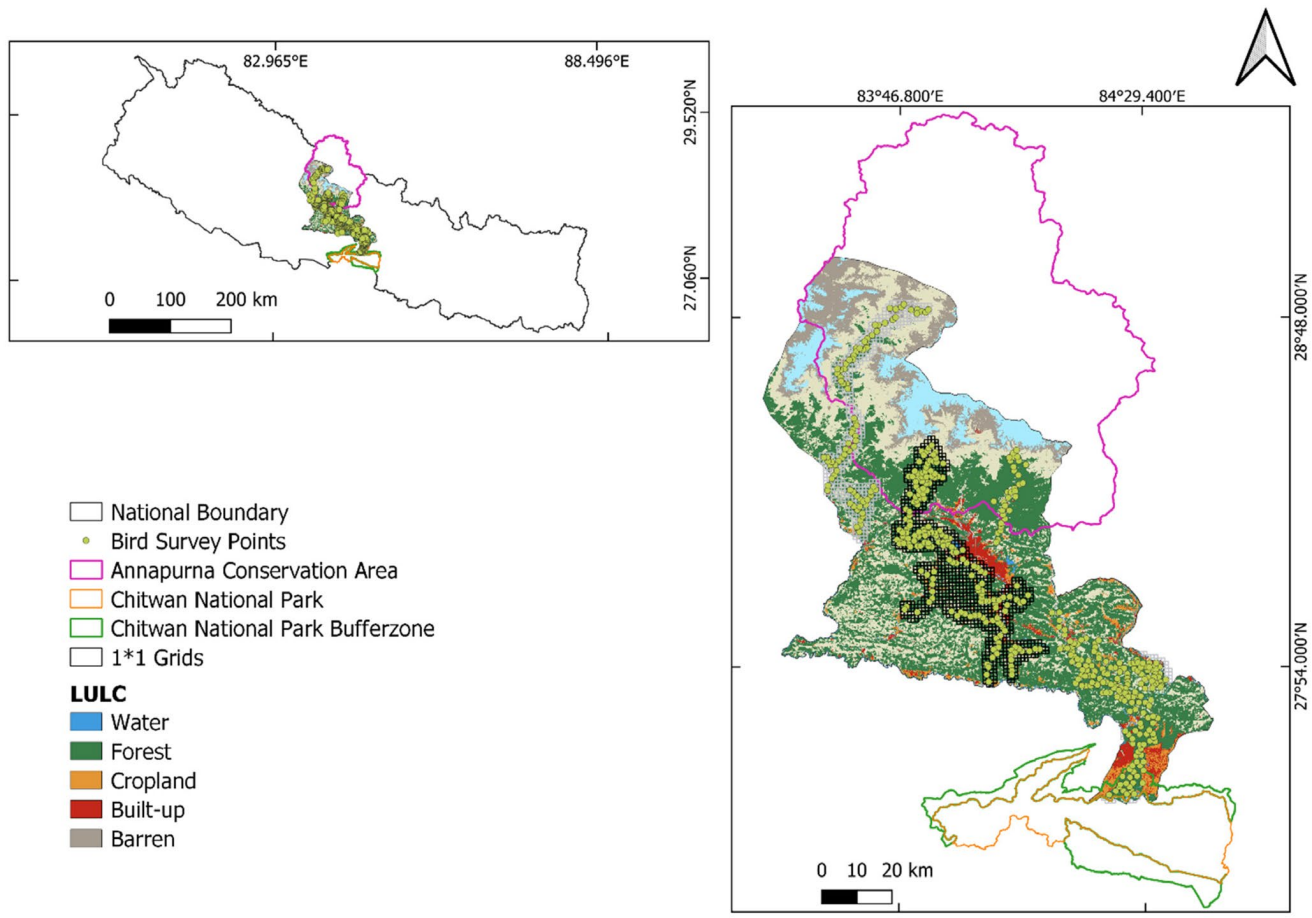
GIS (Geographic information system) map showing bird survey points and Land use and Land Cover (LULC) in the CHAL.

CHAL was designed to maintain and conserve ecological connectivity across Nepal's Himalayan eco‐physiographic zones, extending from Chitwan National Park in the south to Manaslu, Langtang, and Annapurna in the north (Ministry of Forests and Soil Conservation 2015). It is situated in central Nepal, spanning latitudes 27°35′–29°33′ N and longitudes 82°88′–85°80′ E. The CHAL region encompasses all or parts of 19 districts (Mustang, Manang, Gorkha, Rasuwa, Nuwakot, Dhading, Lamjung, Tanahu, Syangja, Kaski, Palpa, Parbat, Baglung, Myagdi, Gulmi, Arghakhanchi, Makwanpur, Chitwan, and Nawalparasi) (Ministry of Forests and Soil Conservation 2015). The landscape of CHAL can be categorized based on elevation, geology, and climate into five zones: Terai (59–200 masl), Siwaliks (200–1500 masl), mid‐hills or mountains (1000–2500 masl), high mountains (2200–4000 masl), and high Himalayas (above 4000 masl). The current study encompasses six districts (Chitwan, Tanahu, Kaski, Parbat, Myagdi, and Mustang) and eight major river systems, including the Kali Gandaki, Seti, Madi, Marshyangdi, Daraudi, Budi Gandaki, Trishuli, and Rapti rivers, along with their tributaries (Figure 1). CHAL encompasses three eco regions from the Global 846 Ecoregions: the Terai‐Duar Savanna and Grasslands, the Himalayan Subtropical Broadleaf Forests, and the Himalayan Subtropical Pine Forests (Olson et al. 2001).

CHAL has the major natural ecosystems such as forests, grasslands and rangelands, wetlands/rivers, and cryosphere (Ministry of Forests and Soil Conservation 2015). Mid‐hills of the CHAL are human‐dominated and represent an extensive anthropogenic matrix of land uses (Adhikari, Bhattarai, et al. 2022; Adhikari, Khatiwada, et al. 2022). However, the forest cover present in the CHAL is highly fragmented.

The forests of CHAL are managed under various regimes, including: (1) protected areas, (2) government‐managed forests, (3) protection forests, and (4) community‐based management systems, such as community forests, leasehold forests, and buffer zone community forests (Ministry of Forests and Soil Conservation 2015). Community forests present in the CHAL play an important role in biodiversity conservation and addressing climate vulnerabilities. Twenty‐nine percent of the forests in the landscape are under the three community‐based management systems (Ministry of Forests and Soil Conservation 2015).

### 2.2 Methods

A pilot survey was conducted in the study area in October 2019 before the data collection to establish baseline data on the habitats, topography, ecology, threats, and bird species. A pilot or preliminary study is conducted before main research to test the feasibility, design, and methodology. It also helps to identify the potential issues, refine data collection, and ensure the reliability and validity of the research.

#### 2.2.1 Data Collection

The line transect method is effective in terrestrial habitats of plain areas; however, we used the grid‐based point count method. In terrestrial habitats across elevation gradients, factors such as uneven terrain, patchy vegetation, and other landscape features can make it difficult to maintain a consistent transect line (Buckland et al. 2001). Therefore, point counts allow for strategic sampling of diverse microhabitats, making them particularly suitable for human‐dominated landscapes like CHAL. The entire study area was divided into 9440 grids of size 1 × 1 km^2^. Altogether, 514 grids were selected by using sample size determination at a 98% confidence level, and a 5% margin of error. Among 514 grids, 472 were selected for the study due to inaccessibility to visit the rest of the grids. In each grid, bird species along with environmental variables were recorded within a 50 m radius. Bird survey was carried out in winter (November–February) and summer (April–July) seasons of the years 2020 and 2021 to cover residential and migratory (i.e., winter and summer) bird species. We spent total of 108 days in the field, including 54 days (27 days in each season) in 2020 and 54 days in 2021. Total man hours spent in the field were 648.

The birds were observed visually using binoculars (Olympus, 20 × 50 magnification) and photographed with a Nikon camera (80× zoom). The point count method was employed to cover a wide range of bird species, enhance detection, and improve identification accuracy (Ralph et al. 1995). A global positioning system (GPS) was used to record the geographic coordinates of the sampling points. Direct observation was conducted to identify and record bird species during their peak activity periods—early morning (7:00 AM–10:00 AM) and late afternoon (3:00 PM–6:00 PM). These time windows were chosen as they correspond to increased feeding activity, with mornings offering optimal foraging conditions as the sun warms up insects, and evenings providing a final opportunity for birds to gather energy before nightfall. During data collection, 1–5 min were allocated for adjustment to the location, followed by 20 min dedicated to counting birds, observing their habitats, and noting other environmental factors. Two observers were involved to report the birds from two directions. This minimizes the chance of missing the bird's species. Careful observation, spatial awareness, temporal considerations, distance between the two sampling points, and observer expertise help to avoid duplicate counting of the birds. The distance between two sampling points was at least 1 km, hence, this minimizes the chances of duplication. The list of the two observers then merged as a single recording. Collected bird's data along with the number of individuals reported, time, and weather conditions by developing a standard data sheet. The calls of the birds were recorded using a recording device for further analysis. The birds were identified by using a field guide book “Birds of Nepal (Grimmett et al. 2016)”. We also consulted with birds' experts for the confirmation of birds by using photographs. We also used the Merlin Bird ID app to identify birds by comparing their photographs and sounds.

#### 2.2.2 Habitat Types

Nepal has a diverse range of ecosystems due to its varied topography and climatic conditions, ranging from the lowland Terai to the high Himalayas. The major habitat types include cropland, forest, shrubland, grassland, and wetland, each playing an important role in biodiversity conservation (Kandel et al. 2018).

- *Cropland*: Cropland is primarily found in the Terai, mid‐hills, and valleys, where agriculture is the main livelihood. The fertile plains of the Terai are dominated by paddy, wheat, and maize cultivation, while hill farming involves terraced fields for crops like millet, barley, and potatoes. Agroecosystems also support a variety of biodiversity, including birds (Paudel et al. 2016).
- *Forest*: It covers around 44% of Nepal's land area. All types of forests found in CHAL categorize as forests. Major forest types found in CHAL are Tropical and subtropical forests (Sal forests in the Terai), Temperate forests (Rhododendron, oak, Schima‐Castanopsis Forest, Alnus, and pine in mid‐hills), Subalpine and alpine forests (Fir, birch, and juniper in higher elevations) (Rokaya et al. 2024). Community forests, managed by local users, play a vital role in conservation and sustainable resource use.
- *Shrubland*: Shrublands are transitional habitats found in degraded forests, riverbanks, and high‐altitude zones. Mid‐hill of the study area and degraded land is covered by the shrubland. They are characterized by dense, low‐lying vegetation, including species like Berberis, *Osbeckia stellata* and *Elsholtzia blanda*, and Rhododendron (Paudel et al. 2011).
- *Grassland*: Grasslands in CHAL range from subtropical savannas in the Terai (e.g., Chitwan National Parks) to alpine meadows in the high Himalayas (Mustang and Kaski). Themeda ( *Themeda villosa* ) followed by Narkat ( *Arundo donax* ) are prominent grass species for tall grassland habitat, whereas dubo ( *Cynodon dactylon* ) and Siru ( *Imperata cylindrica* ) cover the ground level. Scattered grass patches found inside the forest help to increase the feeding and breeding ground for the birds. Grasslands in alpine and subalpine regions are crucial for livestock grazing (called pasture lands), but overgrazing and encroachment threaten their ecological balance (CNP 2016; Rokaya et al. 2024).
- *Wetland*: Wetlands, including rivers, lakes, ponds, and marshes, are among the most ecologically significant habitats for animals. Major wetlands found in the study area are two Ramsar sites—Beeshazari Lake and the Lake cluster of Pokhara Valley, as well as river systems such as Seti, Kaligandaki, Trisuli, Rapti, and Mardi rivers. These areas support rich biodiversity, including migratory birds, fish, amphibians, and aquatic plants (Shrestha et al. 2020).

#### 2.2.3 Physiographic Zones

CHAL is an ecologically diverse region in central Nepal, spanning from the lowland Terai to the high Himalayas. It encompasses a wide range of altitudinal and climatic gradients, resulting in distinct physiographic zones (Paudel et al. 2011; Dhital and Dhital 2015):

- *Terai and Siwalik Hills (Lowland Plains and Churia Range) (150–1500 m)*: Located in the southernmost part of CHAL, the Terai consists of flat, fertile alluvial plains. It is dominated by tropical and subtropical forests, primarily Sal ( *Shorea robusta* ) forests and grasslands. Siwalik is the first range of foothills north of the Terai, composed of fragile sedimentary rocks prone to erosion and landslides. The vegetation consists of dry deciduous forests, including Sal, Chir pine (*Pinus roxburghii*), and riverine species.
- *Middle Hills (Mahabharat Range) (1500–3000 m)*: Characterized by steep terrain, terraced farming, and human settlements, this zone is dominated by forests of oak, rhododendron, and pine species.
- *High Mountains (3000–4000 m)*: A cold temperate to subalpine zone has mixed forests transitioning into alpine meadows. Vegetation includes rhododendrons, junipers, fir, and birch forests.
- *High Himalayas (Above 4000 m)*: A region of harsh alpine and glacial landscapes with extreme climatic conditions. Vegetation is sparse, mainly consisting of alpine grasslands and dwarf shrubs.

### 2.3 Data Analysis

To analyze the composition and diversity of birds, we employed several indices, including Shannon diversity, Pielou's evenness, the Simpson diversity index, species distribution curves, species accumulation curves, and upset plots. Species richness was determined as the total number of species recorded in each season, while abundance referred to the total number of individual birds observed. These metrics, along with diversity indices and species accumulation curves, were calculated using the *vegan* package in R (Oksanen et al. 2013).

Shannon–Wiener's diversity index (*H*′) and Pielou's evenness (*J*) were used to assess bird species composition across seasons and habitats. The Shannon–Wiener index (*H*′) was calculated as: 𝐻′ = −Σ𝑃𝑖 × 𝐿𝑁 (𝑃𝑖).

Here, “*Pi*” represents the proportion of each species in the sample, and “*LN*(*Pi*)” denotes the natural logarithm of this proportion (Hutcheson 1970; Shannon 1948).

The evenness was calculated as: 𝐽 = 𝐻′/ln (𝑆).

Where “*H*” represents Shannon–Wiener's diversity index and “*S*” denotes species richness (Heip 1974). A Whittaker plot was constructed by plotting species abundance against their respective ranks. Additionally, a relative rank abundance curve (RAC) was utilized to analyze species composition and diversity within the community (Avolio et al. 2019).

Non‐metric multidimensional scaling (NMDS) ordination, based on Bray‐Curtis dissimilarities of square‐root transformed species abundance data, was employed to examine spatial and temporal variations in bird community structure. The analysis was conducted using the BiodiversityR package, followed by an analysis of similarity (ANOSIM) test.

The Detrended Correspondence Analysis (DCA) revealed a gradient length of 10.8556, confirming the suitability of Canonical Correspondence Analysis (CCA) for the data (Table 1). To evaluate the significance of the ordination with respect to environmental variables, a Monte Carlo permutation test with 999 iterations was conducted. Additionally, species overlap was visualized using an upset plot generated with the UpSetR package. All analyses were performed in R Studio (R Core Team 2024).

**TABLE 1:** Detrended correspondence analysis (DCA) results showing whether unimodal or linear ordination methods were appropriate.

	DCA1	DCA2	DCA3	DCA4
Eigenvalues	0.7587	0.5726	0.5733	0.5439
Additive Eigenvalues	0.7587	0.5699	0.5741	0.5383
Decorana values	0.894	0.7577	0.6714	0.6232
Axis lengths	10.8556	9.7517	9.28	7.6318

## 3 Results

### 3.1 Species Richness, Diversity and Composition

A total of 11,653 individuals were recorded during the study, representing 20 orders, 77 families, and 458 species across the CHAL. Out of 458 species, a higher number of bird species were recorded in winter (160 species) than in the summer season (96 species), and the remaining 202 species were reported in both seasons. This study reported three globally critically endangered species ( *Gyps bengalensis* , *Gyps tenuirostris* , *Sarcogyps calvus* ), two globally endangered species ( *Falco cherrug* , *Neophron percnopterus* ), two globally vulnerable species ( *Leptoptilos javanicus* , *Prinia cinereocapilla* ), and five globally near‐threatened species ( *Gypaetus barbatus* , *Gyps himalayensis* , *Icthyophaga ichthyaetus* , *Psittacula alexandri* , *Vanellus duvaucelii* ) (Table S1). This study also reported five nationally critically endangered species ( *Gyps bengalensis* , *Gyps tenuirostris* , *Haliaeetus albicilla* , *Icthyophaga ichthyaetus* , *Prinia cinereocapilla* ) and four nationally endangered species ( *Falco cherrug* , *Harpactes erythrocephalus* , *Ibidorhyncha struthersii* , *Sarcogyps calvus* ).

This study indicates the high diversity indices (Shannon = 5.54, Simpson = 0.99) with equal species representation and low dominance. Pielou's Evenness Index was 0.90, which indicates high evenness in this study area (Table 2).

**TABLE 2:** Overall richness, abundance, diversity, and evenness of birds in CHAL.

Parameters	Value
Species richness	458
Total abundance	11,653
Shannon diversity	5.54
Margalef richness index	48.7
Simpson diversity	0.99
Pielou's evenness	0.9

**FIGURE 2:**
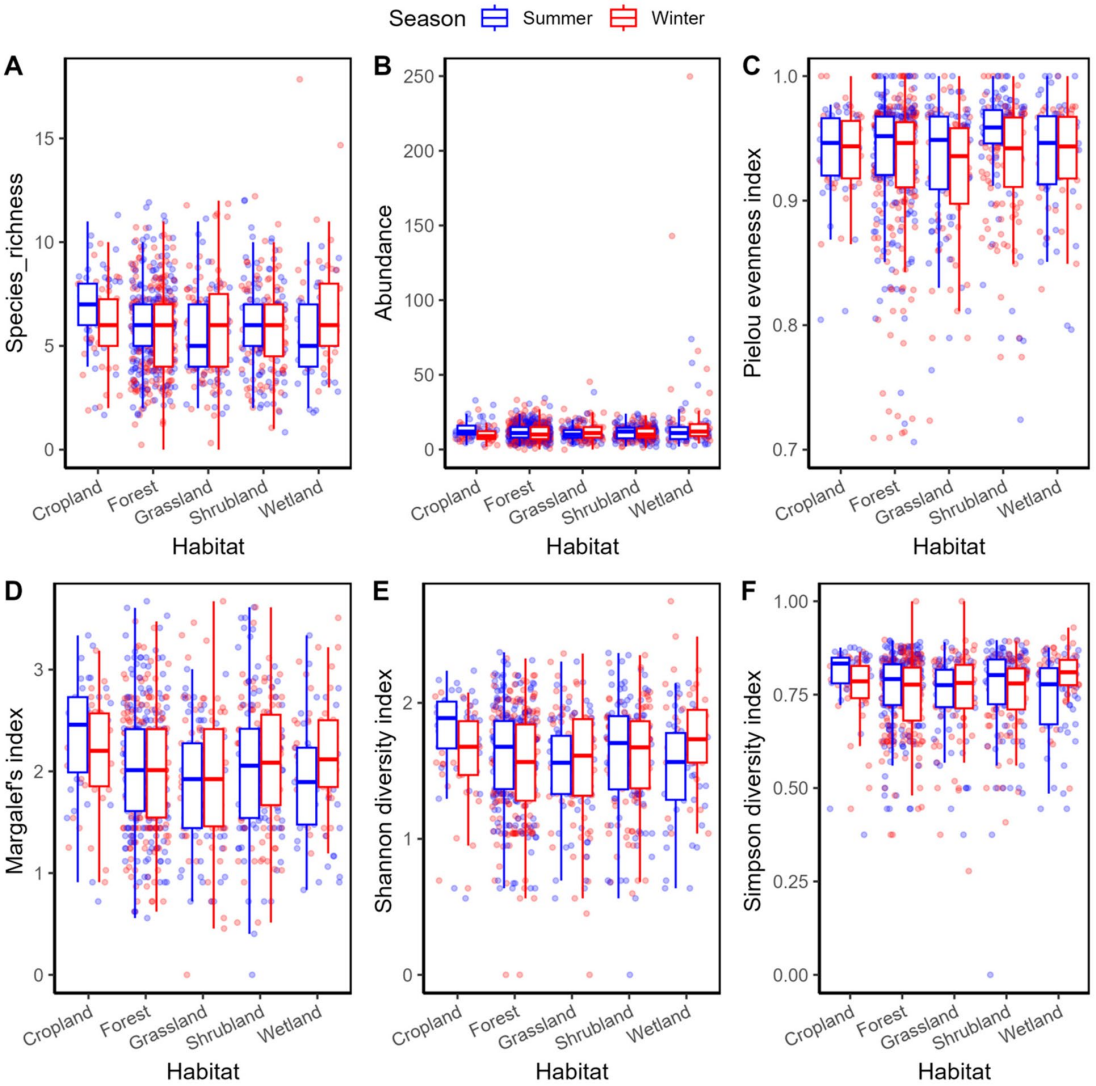
Boxplot showing bird species richness, abundance, evenness, Shannon, and Simpson index of diversity in the study areas.

Species richness (Figure 2A) patterns revealed interesting habitat‐specific responses to seasonal changes. Wetland habitat showed the most dramatic seasonal variation, with winter richness (6.94 ± 0.38) higher than summer (5.55 ± 0.28). Cropland exhibited similar seasonal patterns but in the opposite direction, with higher richness in summer (7.00 ± 0.35) compared to winter (5.86 ± 0.32). Forest habitat maintained intermediate richness values with minimal seasonal variation (summer: 6.00 ± 0.14; winter: 5.67 ± 0.15), suggesting a more stable community structure.

Abundance (Figure 2B) was higher in summer than in winter across cropland, shrubland, and forest, while grassland and wetland had higher abundance in winter. Wetland habitat demonstrated the highest mean abundance, particularly in winter (23.10 ± 5.65), showing a dramatic seasonal increase from summer (14.08 ± 1.89). Cropland exhibited the second‐highest summer abundance (13.61 ± 1.10) but showed a marked decrease in winter (10.03 ± 0.82). Forest habitat maintained a relatively stable abundance across seasons (summer: 12.21 ± 0.37; winter: 11.28 ± 0.41).

Pielou's evenness index (*J*) (Figure 2C) exhibited high values across all habitat types, ranging from 0.90 ± 0.01 to 0.95 ± 0.00, indicating relatively equal species distributions in all studied communities. Shrubland showed the highest summer evenness (0.95 ± 0.00), while Grassland demonstrated the lowest winter evenness (0.90 ± 0.01). Seasonal fluctuations in evenness were minimal across most habitat types (∆*J* < 0.05). This suggests overall temporal stability in community structure across the study area.

Margalef's diversity index (Figure 2D) showed the highest values in cropland during summer (2.33 ± 0.10), followed by wetland in winter (2.19 ± 0.08). Forest habitat maintained intermediate values with minimal seasonal variation (summer: 2.02 ± 0.04; winter: 1.99 ± 0.04), while grassland consistently showed lower values (summer: 1.88 ± 0.07; winter: 1.97 ± 0.08). The Shannon diversity index (Figure 2E) showed highest values in cropland during summer (1.77 ± 0.06) and wetland during winter (1.75 ± 0.05), indicating these habitats support the most diverse communities in their respective peak seasons. Forest habitat maintained moderate diversity levels across seasons (summer: 1.62 ± 0.03; winter: 1.53 ± 0.03), while grassland showed the most consistent diversity between seasons (summer: 1.52 ± 0.04; winter: 1.54 ± 0.05). Simpson diversity (Figure 2F) patterns largely paralleled Shannon diversity, with the highest values in cropland during summer (0.80 ± 0.02) and wetland during winter (0.79 ± 0.01). This consistency across diversity metrics strengthens the reliability of these findings.

These curves illustrate the relationship between sampling effort and observed species richness, with information on biodiversity patterns and sampling adequacy. Winter consistently shows higher species richness compared to summer across all sampling efforts, indicating seasonal variation in bird diversity. Among habitats, forest areas support the highest species richness, followed by grassland, wetland, shrubland, and cropland. Similarly, species richness varies across physiographic zones, with the Terai and Siwalik region exhibiting the highest diversity, followed by the Middle Hills, High Mountain, and High Himalaya regions.

**FIGURE 3:**
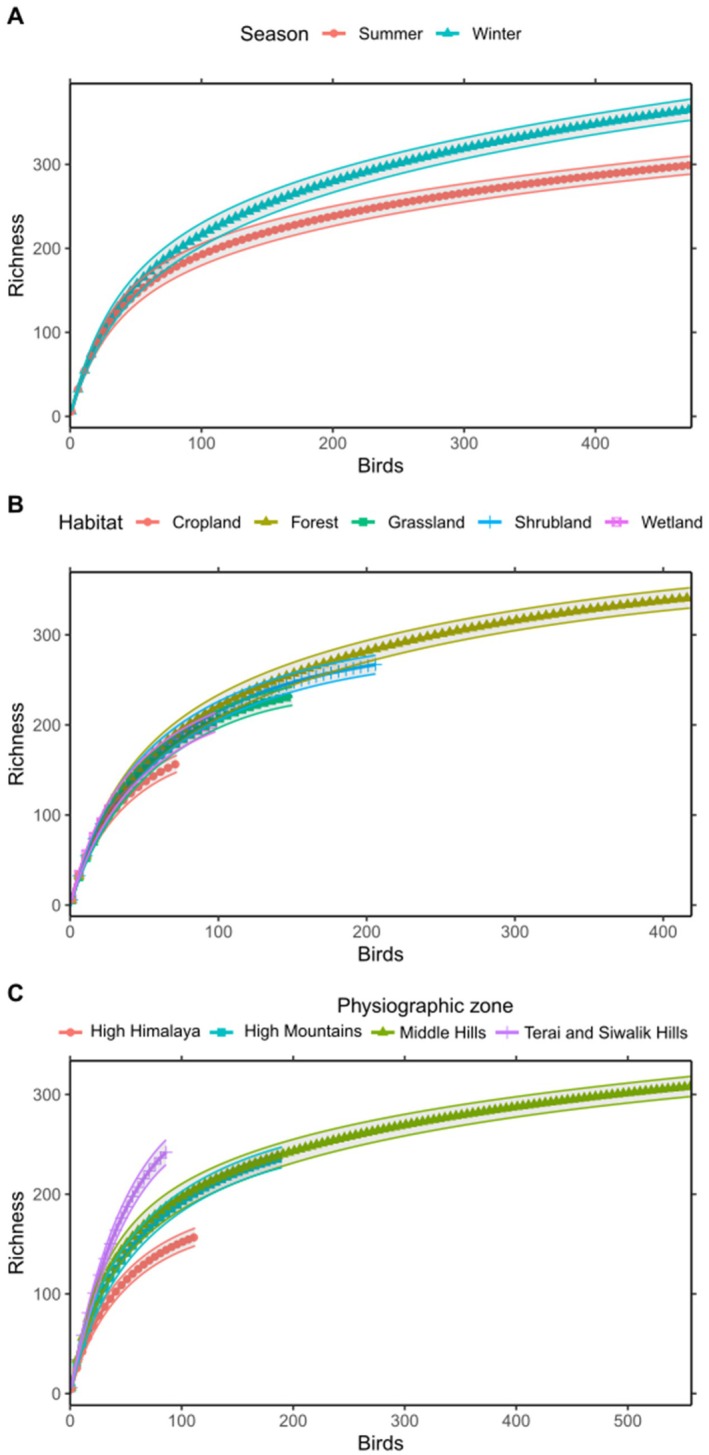
Species accumulation curve for birds in (A) season, (B) habitats, and (C) physiographic zones.

Despite the clear patterns observed, the consistent upward trends in some variables indicate that the bird species' lists in the region are not yet saturated. Additional sampling efforts are likely to yield new species records (Figure 3).

**FIGURE 4:**
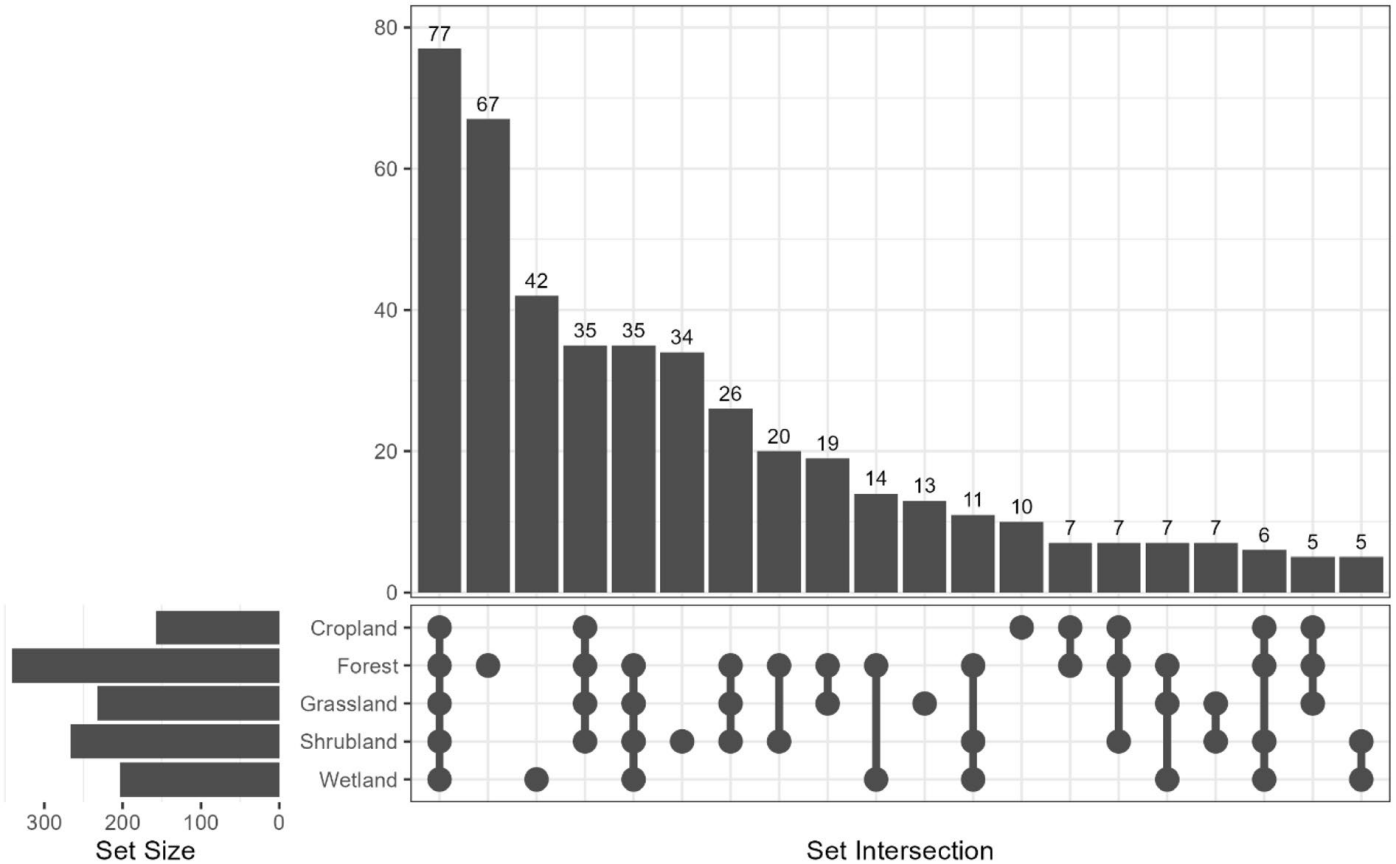
Upset plot showing the number of species in specific habitats and the intersection of species distribution across different habitats.

The plot provides a visual representation of the distribution and overlap of species across the studied habitats (Figure 4). The set size plot represents the total number of species recorded in each habitat. The highest number of species was found in forest, followed by shrubland, grassland, wetland, and cropland. The set intersection plot displays the size of species intersection for combinations of habitats. The connecting dots in the intersection plot represent shared species among habitats, while a single dot signifies species exclusive to a particular habitat.

The first column reveals that 77 species were present across all five habitat types, representing the highest overlap. The second column shows that 67 species were exclusively found in forests. Additionally, 42 species were found only in wetlands, 34 species in shrublands, 13 species in grasslands, and 10 species in croplands.

**FIGURE 5:**
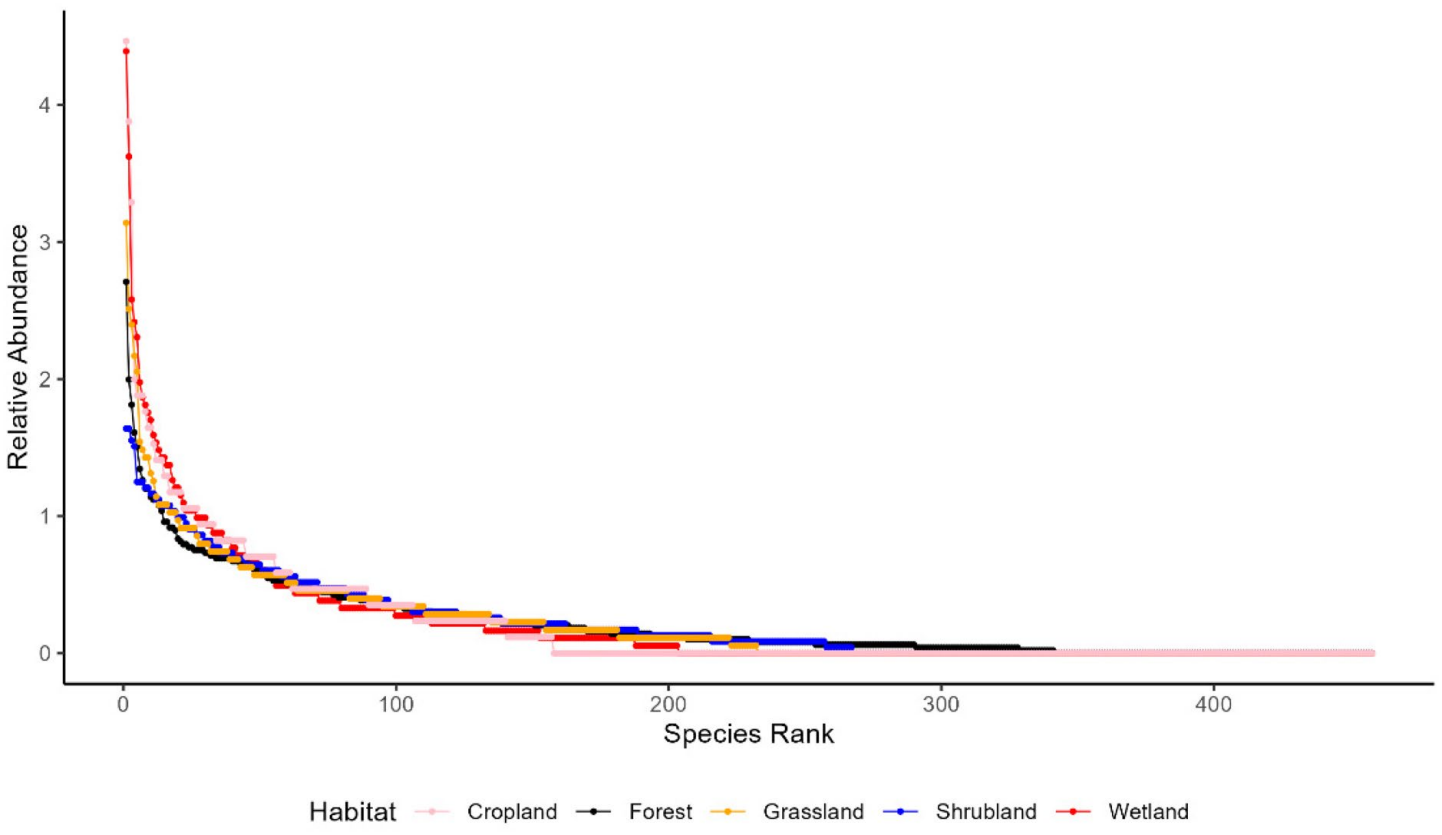
Relative rank abundance distribution curve of different habitats.

A total of 458 species recorded in CHAL were categorized into 325 distinct ranks based on the number of individuals observed for each species. The steep gradient highlights low evenness, as high‐ranking species indicate significantly greater abundances compared to those ranked lower. Conversely, a gentle slope indicates higher evenness, where species abundances are more evenly distributed. Notably, the RAC for grassland and forest was higher than that for wetlands and open areas (Figure 5).

### 3.2 Community Composition

The NMDS ordination, used to visualize the variation in bird community composition, yielded a stress value of 0.05. It indicates a reliable representation of the underlying ecological relationships in two dimensions and reveals complex patterns in avian community structure across multiple ecological dimensions: seasonal variation, physiographic zones, and habitat types.

**FIGURE 6:**
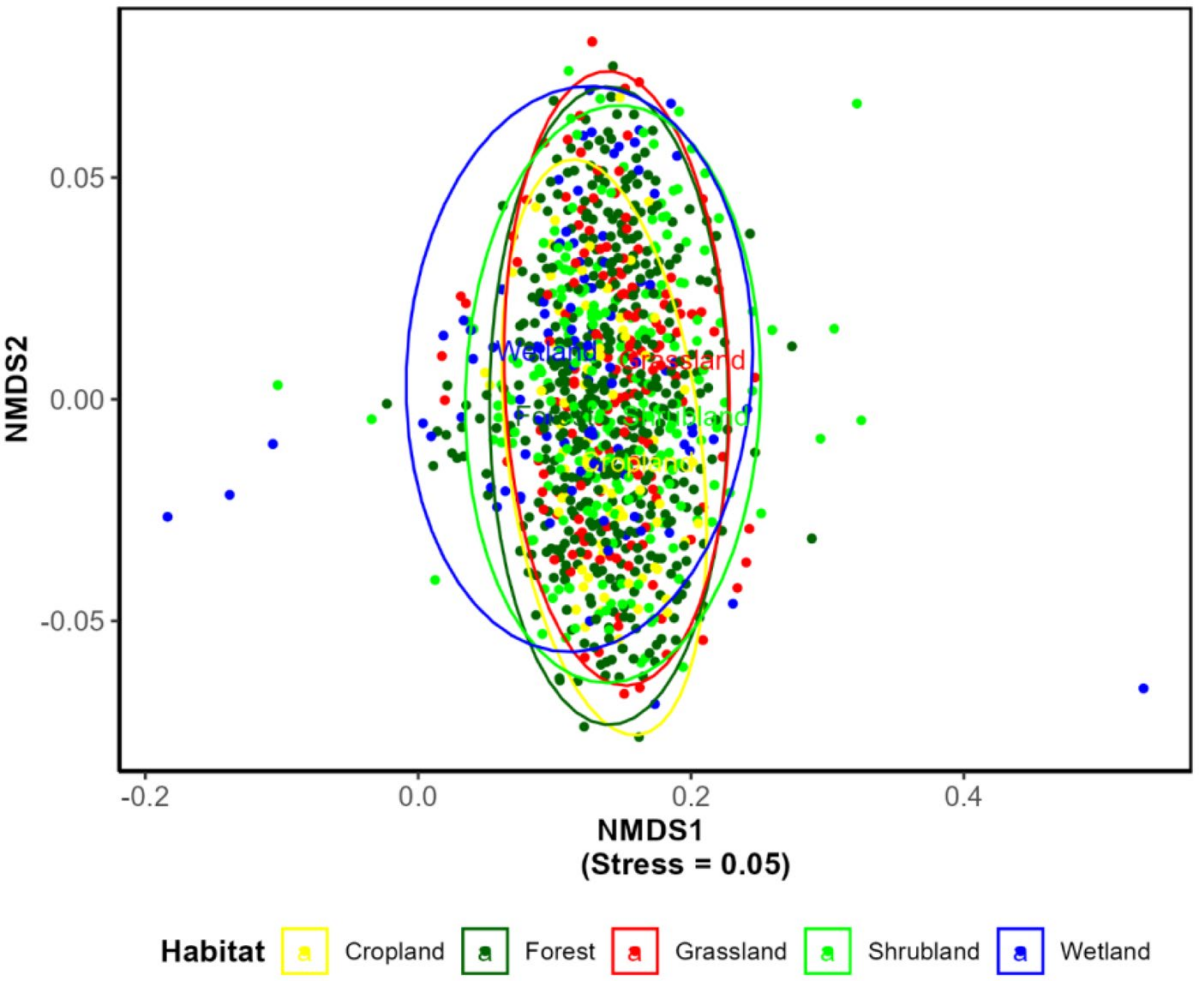
NMDS showing the community composition of birds in different habitats.

The ordination plot demonstrates considerable overlap among habitat types, suggesting a degree of similarity in bird assemblages across different habitats (Figure 6). However, wetland communities show some distinction, particularly at the ordination periphery, indicating a distinct bird assemblage associated with aquatic environments. Forest and shrubland communities display overlap in ordination space, suggesting similar bird community composition. Cropland and grassland sites show intermixing in the central portion of the ordination space, indicating similar bird communities. Some common birds such as Intermediate Egret (*Ardea intermedia*), Indian Pond Heron ( *Ardeola grayii* ), Cattle Egret ( *Bubulcus ibis* ), Northern Goshawk ( *Accipiter gentilis* ), Paddyfield Pipit ( *Anthus rufulus* ), Steppe Eagle ( *Aquila nipalensis* ), White Wagtail ( *Motacilla alba* ), Yellow Wagtail ( *Motacilla flava* ), Egyptian Vulture ( *Neophron percnopterus* ), Oriental Magpie Robin ( *Copsychus saularis* ), etc., were reported from all types of habitats (Table S1).

**FIGURE 7:**
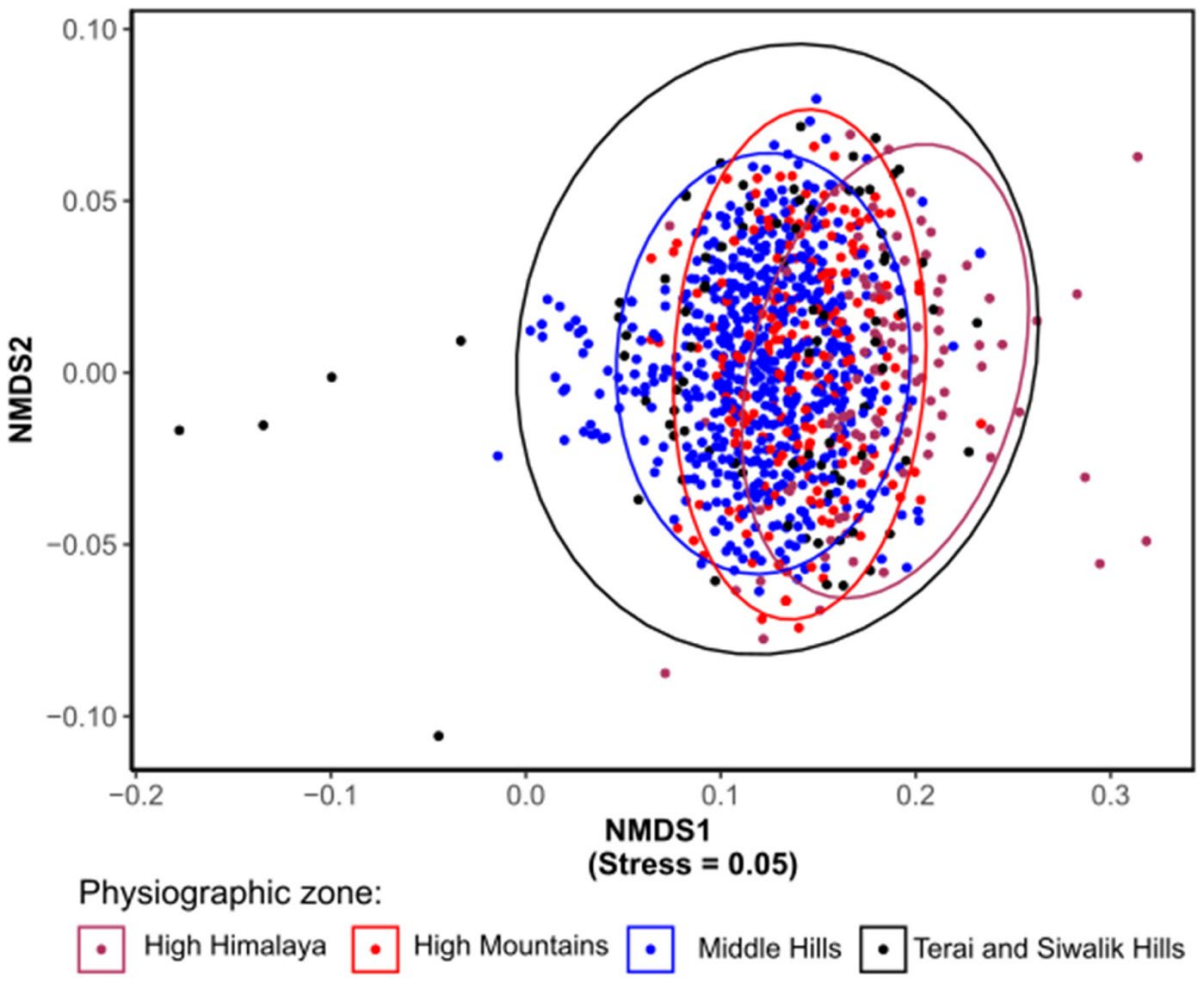
NMDS showing the community composition of birds in different physiographic zones.

The analysis across physiographic zones (Terai and Siwalik Middle Hills, High Mountains, and High Himalaya) shows overlap, particularly among Middle Hill and High Mountain communities (Figure 7). This overlap suggests that elevation gradients may not be the primary driver of community assembly. However, the slight separation of Terai and Siwalik communities (particularly at the ordination edges) shows distinct lowland assemblages. Some bird species such as Cinereous Vulture ( *Aegypius monachus* ), Mallard ( *Anas platyrhynchos* ), Common Moorhen ( *Gallinula chloropus* ), White‐rumped Vulture ( *Gyps bengalensis* ), Slender‐billed Vulture ( *Gyps tenuirostris* ), Ibisbill ( *Ibidorhyncha struthersii* ), Scaly‐breasted Munia ( *Lonchura punctulata* ), Egyptian Vulture ( *Neophron percnopterus* ), Blue‐capped Redstart ( *Phoenicurus coeruleocephala* ), Common Stonechat ( *Saxicola torquatus* ), Black Redstart ( *Phoenicurus ochruros* ), Red‐headed Vulture ( *Sarcogyps calvus* ), etc. were reported from all types of physiographic zones.

**FIGURE 8:**
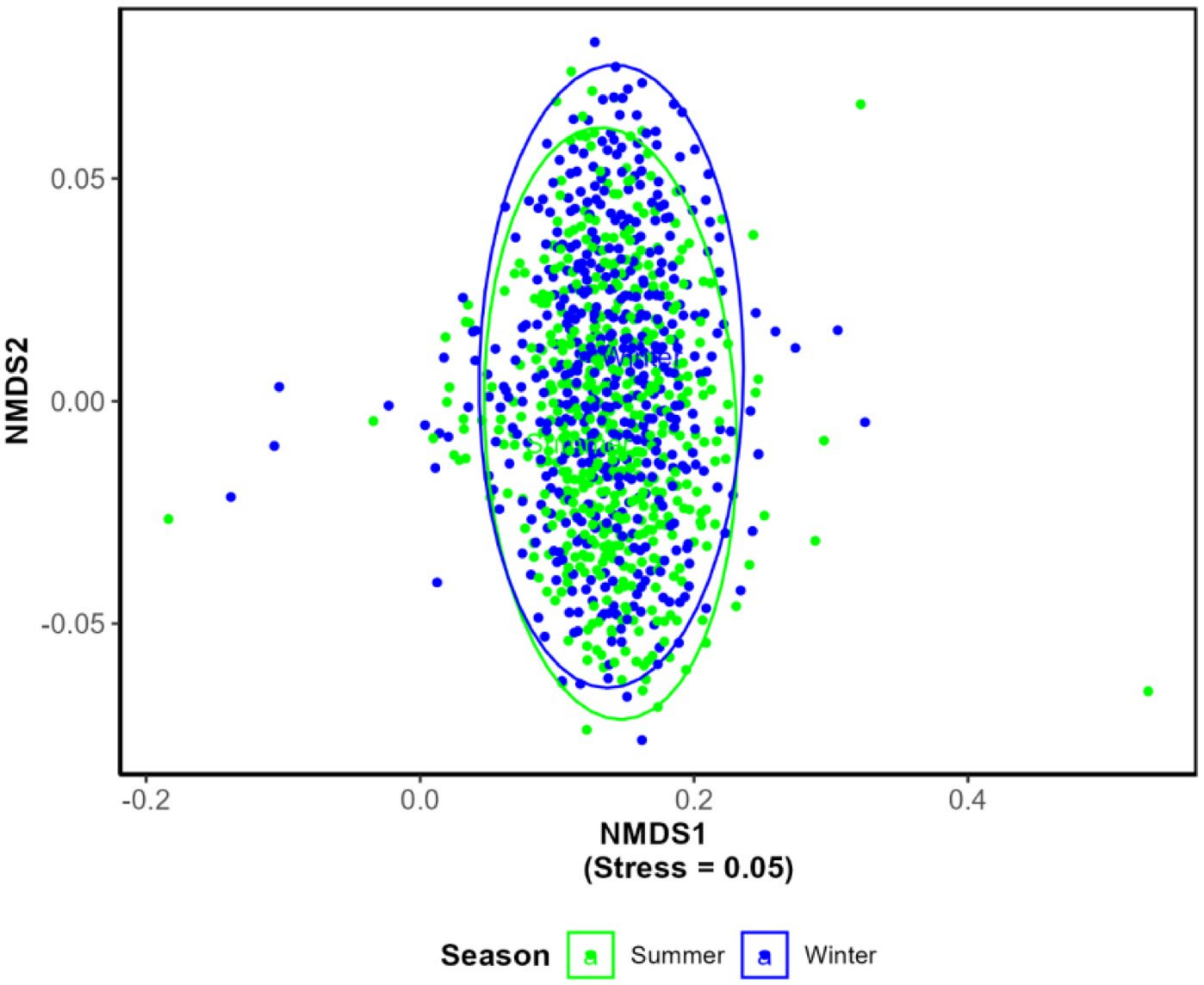
NMDS showing the community composition of birds in different seasons.

**FIGURE 9:**
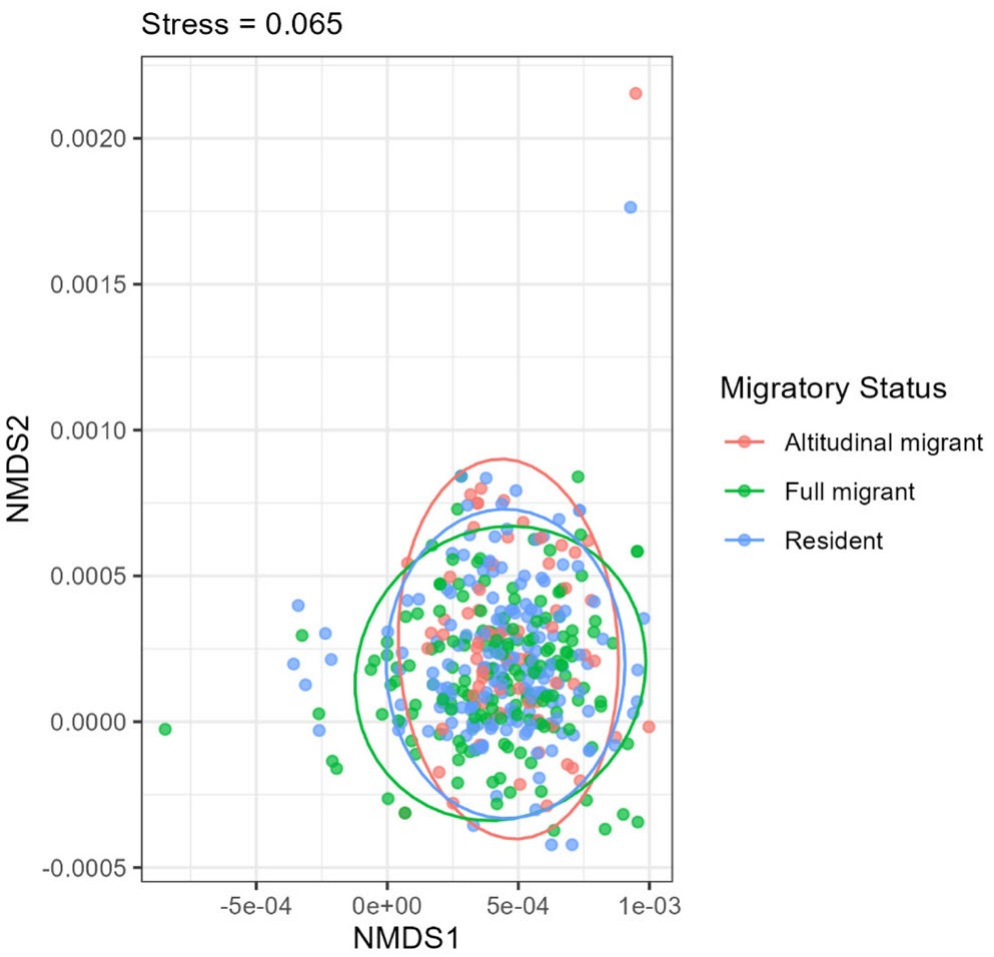
NMDS showing the community composition of birds in migratory and residential status.

The ordination shows a significant overlap between summer and winter bird communities, indicating a core group of resident birds (Figure 8). However, the slight differences in the shapes and positions of the ellipses indicate seasonal changes in species. This reflects a mix of resident and migratory birds, with winter having slightly more variation in community composition. The NMDS plot (Figure 9) illustrated that bird communities in the study area were generally composed of a blend of resident, altitudinal migrant, and full migrant species. While there might be variations in the proportions of these groups in certain locations (indicated by the peripheral points), migratory status alone does not strongly differentiate the overall bird community composition across the sampled areas. This highlights the complexity of the avian community. To further clarify the compositional variation along these factors, ANOSIM was carried out.

### 3.3 Analysis of Similarity

ANOSIM results revealed varying degrees of community differentiation across different ecological parameters, with all parameters showing statistically significant differences (*p* < 0.01) (Table 3). The strongest community segregation was observed across physiographic zones (*R* = 0.09, *p* = 0.001), suggesting that elevation and associated environmental gradients play a substantial role in structuring bird communities. However, the relatively low *R*‐value indicates considerable overlap between zones. Seasonal variation showed moderate community differentiation (*R* = 0.06, *p* = 0.001). Habitat type showed the weakest, though still significant, effect on community structure (*R* = 0.01, *p* = 0.007). This low *R*‐value indicates a high degree of species sharing between habitat types and possible habitat generalist dominance.

**TABLE 3:** Analysis of similarity (ANOSIM) presents community differentiation across different ecological parameters.

S. No.	Parameters	*R*‐statistics	*p*
1	Season	0.06	0.001
2	Habitat	0.01	0.007
3	Physiographic zone	0.09	0.001

**FIGURE 10:**
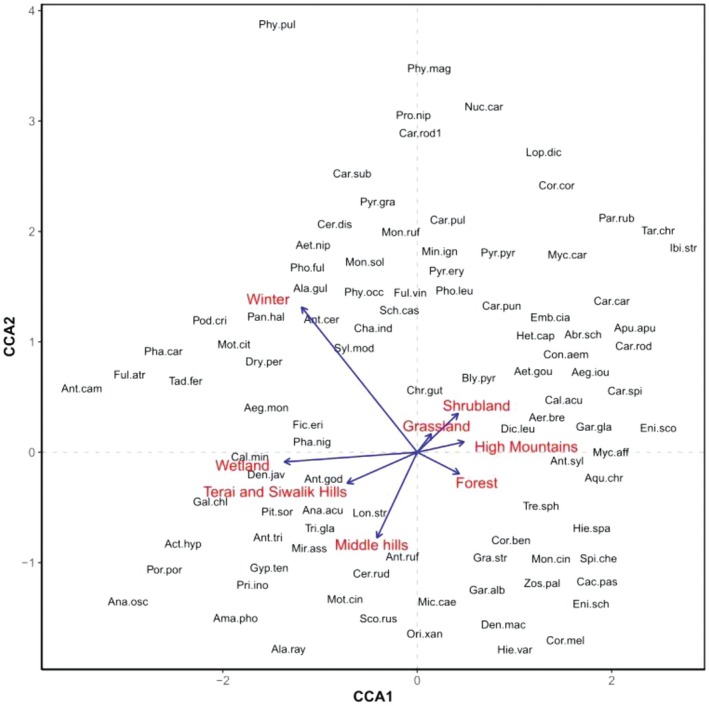
CCA plot showing the correlation between bird species, habitats, physiographic zone, and season.

The CCA ordination plot depicts the alignment of bird species with environmental variables, including season, habitat types, and physiographic zones. The analysis demonstrates clear habitat segregation along both CCA axes, with forest, shrubland, wetland, and grassland habitats showing distinct clustering (Figure 10). However, there is notable overlap between some habitat types. The physiographic zones (Mountain, Hill, Siwalik) appear to exert a strong influence on species distribution. However, the relatively short length of some environmental vectors suggests that their explanatory power might be limited. The winter and wetland habitats have a major contribution to the variance in the bird's species associations. *Ficedula westermanni* , *Calliope calliope*, and *Phoenicurus fuliginosus* show more association with the winter season. Similarly, bird species like ( *Actitis hypoleucos* , *Dendrocygna javanica* ) show a stronger association with wetland habitat.

## 4 Discussion

Our study provides detailed information on bird species richness, diversity, and community composition across various habitats in the CHAL. A total of 11,653 individuals representing 458 species were recorded, highlighting the rich avian biodiversity in the region. Our findings reveal seasonal, habitat‐related, and different physiographic range variations in species richness and community composition. The diversity patterns observed in this study, as reflected by Margalef's, Shannon's, and Simpson's indices, highlight the influence of habitat type and seasonality on avian community structure. These findings suggest that cropland and wetland habitats exhibit the highest seasonal peaks in bird diversity, while forest and grassland habitats maintain more stable diversity levels across seasons. High Pielou's evenness values across all habitats suggest a stable community that may be due to the absence of strong dominance by any particular species, as well as the effective resource partitioning across different ecosystems.

The CHAL is a biologically rich, climatically and ecologically diverse region that extends a wide altitudinal gradient—from the tropical lowlands of the Terai to the alpine zones of the Himalayas (Adhikari et al. 2024). This landscape is characterized by a mosaic of interconnected habitats, including tropical and subtropical forests (Sal forests, riverine forest in the Terai), temperate forests (Rhododendron, oak, Schima‐Castanopsis Forest, Alnus and pine in mid‐hills), subalpine and alpine forests (Fir, birch, and juniper in higher elevations) (WWF 2013; Rokaya et al. 2024). Besides these, grasslands, croplands, and shrublands present in different altitudinal areas mosaic the fragmented and scattered habitats of CHAL. These habitats are not isolated; even though they are human‐dominated and fragmented, they form an ecological continuum that allows for the movement and interaction of species across different environmental zones (Adhikari et al. 2024). This interconnected landscape is important for birds. The resident birds can move locally with the seasons for food, altitudinal migrants travel up and down mountains for food and breeding, and full migrants pass through on their long journeys (WWF 2013).

The bird community in any habitat undergoes seasonal changes (Avery and van Riper III 1989). The seasonal variations in species diversity are linked to the availability of resources, climatic conditions, and seasonal migration patterns (Katuwal et al. 2016; Pandey et al. 2020). Therefore, seasonal variation has a significant impact on the species richness and distribution of birds (Cueto and Lopez de Casenave 2000). In Nepal, it plays a crucial role in shaping the composition of bird species (Grimmett et al. 2016; Inskipp et al. 2016a, 2016b; Katuwal et al. 2016). In this study, species richness was generally higher in winter than in summer across most habitat types, except ‘Cropland’ habitat. The general increase in winter richness could indicate that many habitats serve as important winter refuges like (Cinereous Vulture *Aegypius monachus* , Yellow‐bellied Fantail *Chelidorhynx hypoxanthus*, Red‐throated Flycatcher *Ficedula albicilla* ) that show a wide range of habitats and altitudinal gradients or that winter conditions in this area favor species diversity, possibly due to resource availability or reduced competition. Another reason is that during the summer season, heavy rainfall and flooding in the river can destroy the birds' breeding and feeding grounds. According to Desgranges et al. (2006), several bird species nest at or near the water surface, making them vulnerable to nest flooding. Moreover, during the winter season, increased local movement of birds in search of food, along with the defoliation of plants, makes bird species easier to detect (Tzortzakaki et al. 2017; Katuwal et al. 2018), which may also increase the diversity in winter. Our findings support Caula et al. (2008), Carbó‐Ramírez and Zuria (2011), Katuwal et al. (2018), Poudel et al. (2021), Shah and Sharma (2022) and Adhikari et al. (2023), who found that species richness was higher during the winter season compared to the summer. However, Keten et al. (2020) observed low richness in winter, while Verma and Murmu (2015) recorded high richness in spring, which contrasts with the findings of this study.

Areas with more open or green spaces, farmlands, and fewer impervious surfaces offer heterogeneous habitats, enhancing bird diversity and richness in the region (Verma and Murmu 2015; Ferenc et al. 2016). In our study area, croplands exhibit the highest species richness during the summer season. This could be due to croplands that are surrounded by a mosaic of vegetation types (fodder and fruits) provide many opportunities for bird foraging and nesting (Jokimäki and Kaisanlahti‐Jokimäki 2012). Fodder plants like *Artocarpus lakoocha* (Badahar), Ficus species (*Ficus semicordata*, *Ficus roxburghii*, and *Ficus lacor*), *Bauhinia purpurea* (Tanki), *Garuga pinnata* (Dabdabe), *Litsea polyantha* (Kutmiro), *Morus alba* (Kimbu), *Grewia optiva* (Phorso); and fruiting plants such as (Citrus species‐ *Citrus reticulata* (Mandarin orange), Kagati (lemon), and other citrus fruits), *Choerospondias axillaris* (Lapsi), *Musa paradisiaca* (Banana), *Mangifera indica* (Aamp), *Litchi chinensis* (Litchi), *Carica papaya* (Papaya), *Psidium guajava* (Guava), *Pyrus serotina* (Pear), and *Prunus persica* (Peach) are commonly planted surrounding croplands in lowland and mountainous regions of Nepal (Acharya 2006; Upreti and Devkota 2017; Chitale et al. 2018).

Forest, shrubland, and grassland show more stable habitat than others in terms of richness. Vegetative covers provide the species with nesting, roosting, shelter, and foraging, ultimately uplifting the species richness and diversity (Marzluff and Ewing 2008; Menon et al. 2015). These habitats often have more consistent and diverse food resources throughout the year compared to habitats like croplands or wetlands, which might experience seasonal or human‐induced fluctuations in resource availability.

Season was identified as a key factor influencing the abundance and distribution of both migratory and resident birds (Girma et al. 2017). The high abundance of birds in the settlement during winter was due to more food availability in the croplands and settlement areas. Croplands provide plentiful food resources, enhancing breeding success and overall productivity (Sundar 2006; Janiszewski et al. 2014).

In South Asia, birds rely significantly on wetlands and agricultural lands year‐round for breeding and foraging (King et al. 2010; Sundar et al. 2016; Kittur and Sundar 2021). The high increment of bird abundance in settlement as well as in wetland areas during winter may be due to the more availability of food in these habitats than in forest, shrubland, and grassland. The paddy field (cropland) supports many wetland species. Some species such as the Intermediate Egret (*Ardea intermedia*), Indian Pond Heron ( *Ardeola grayii* ), Cattle Egret ( *Bubulcus ibis* ), Northern Goshawk ( *Accipiter gentilis* ), Paddyfield Pipit ( *Anthus rufulus* ), and White Wagtail ( *Motacilla alba* ) are regarded as wetland birds, but they share different types of habitats like forest, grassland, and cropland for foraging. Most of them use grassland and forest for nesting; hence, significant multiple habitats have been seen in these wetland birds (Adhikari, Bhattarai, et al. 2022; Adhikari, Khatiwada, et al. 2022). Wetlands offer abundant food resources, supporting enhanced breeding performance and foraging activities (Sundar 2006; Janiszewski et al. 2014). Additionally, the presence of larger trees maintained by farmers plays a crucial role in supporting various waterbird species (Hilaluddin and Shawl 2003; Koju et al. 2019; Katuwal et al. 2022) and provides them with roosting and nesting sites. During the summer season, rainfall is abundant, and more nutrients are available in the soil, which results in more efficient absorption, increasing leaf development and productivity (Gei and Jennifer 2013; Hulshof et al. 2013). As a result, the abundance of grass and other insects will also be high (Jenber and Wili 2021). Due to this, in summer, the abundance of birds may be high in natural habitats like grassland and trees as compared to winter. These habitats offer species nesting, roosting, shelter, and foraging opportunities, ultimately enhancing their abundance (Marzluff and Ewing 2008; Menon et al. 2015). By summer, the hatching of bird offspring increases the overall population. Juvenile birds add to the abundance, particularly in stable habitats like forests and grasslands where food and shelter are abundant. Migratory and residential waterfowl species play a vital role in wetland ecosystems (Wei and Mundkur 2004). However, during winter, migratory waterfowl and waders arrive in large numbers for overwintering. These species contribute significantly to bird abundance during winter.

The presence of globally and nationally vulnerable species such as *Clanga clanga*, *Clanga hastate*, *Leptoptilos javanicus* , along with endangered species like *Falco cherrug* and near‐threatened species such as *Anhinga melanogaster* and *Vanellus duvaucelii* indicates that CHAL is a suitable habitat for the threatened species.

For the effective conservation of bird species across the diverse elevations and habitats of CHAL, a comprehensive, multi‐faceted approach is crucial. Sustainable forest management practices help maintain forest structure and biodiversity, while proactive grassland management addressing natural succession and controlling invasive species plays a key role in protecting grassland birds. Similarly, the restoration and protection of degraded wetlands enhance their ecological functions. For endangered species like vultures, dedicated species management plans are essential. Additionally, raising community awareness about the importance of bird conservation plays a vital role. Strong collaboration among local communities, NGOs, INGOs, conservation organizations, and government agencies is necessary to ensure the long‐term preservation of birds and their habitats.

## 5 Conclusion

This study underscores the significance of understanding the abundance and seasonal and habitat‐wise diversity of birds in the CHAL, providing valuable insights into the dynamics of species composition across seasons and habitats. The variations in species richness, diversity, and evenness between seasons and habitats highlight the potential influence of seasonality, migration patterns, and habitat dynamics on the composition of CHAL birds. The results highlight the critical role of habitat heterogeneity and seasonality in shaping avian diversity. Croplands and wetlands emerge as bird diversity hotspots during their respective peak seasons. Forests and grasslands, on the other hand, demonstrate greater ecological stability. Long‐term monitoring and regular assessment of bird species are essential to understand the current situation and ensure the long‐term health of the birds in the landscape. Conservation strategies should prioritize the protection and restoration of wetlands and forests, while promoting sustainable agricultural practices to maintain the ecological integrity of croplands.

## Author Contributions

**Shubhas Chandra Bastola:** conceptualization (lead), data curation (equal), formal analysis (equal), funding acquisition (lead), investigation (lead), writing – original draft (equal), writing – review and editing (equal). **Pradip Kandel:** formal analysis (equal), investigation (supporting), writing – original draft (supporting), writing – review and editing (equal). **Hathan Ram Mahato:** investigation (supporting), validation (equal), writing – review and editing (supporting). **Jagan Nath Adhikari:** conceptualization (equal), data curation (equal), investigation (supporting), supervision (supporting), writing – review and editing (equal). **Bishnu Prasad Bhattarai:** conceptualization (lead), data curation (lead), formal analysis (lead), investigation (lead), methodology (lead), project administration (lead), supervision (lead), validation (equal), visualization (lead), writing – original draft (lead), writing – review and editing (lead).

## Conflicts of Interest

The authors declare no conflicts of interest.

## Supporting information


**Table S1.** Checklist of birds of Chitwan Annapurna Landscape, Central Nepal.

## Data Availability

The Supporting Information associated with this manuscript are available at Dryad: https://doi.org/10.5061/dryad.7m0cfxq5n.
