# Supplementary material for: Diversity, Abundance and Community Composition of Birds in Chitwan Annapurna Landscape, Central Nepal
Source: Ecol Evol. 2025 Jul 14;15(7):e71781. doi: 10.1002/ece3.71781 (PMC12259398; doi:10.1002/ece3.71781)
Supplement: Supplementary file 1 — Table S1. Checklist of birds of Chitwan Annapurna Landscape, Central Nepal. [file ECE3-15-e71781-s001.docx]

**Supplementary Table 1.** Checklist of birds of Chitwan Annapurna Landscape, Central Nepal.

| **S.N.** | **Common name** | **Scientific name** | **Code** | **IUCN Redlist** | **NRDB** | **No. of individuals** | **Habitat type** |
| --- | --- | --- | --- | --- | --- | --- | --- |
| **Order: Accipitriformes** | | | | | | | |
| **Family: Accipitridae** | | | | | | | |
| 1 | Shikra | *Accipiter badius* (Gmelin, 1788) | Acc.bad | LC | LC | 25 | Forest, shrubland, grassland, wetland |
| 2 | Northern Goshawk | *Accipiter gentilis* (Linnaeus, 1758) | Acc.gen | LC | LC | 21 | Forest, shrubland, grassland, wetland, cropland |
| 3 | Eurasian Sparrowhawk | *Accipiter nisus* (Linnaeus, 1758) | Acc.nis | LC | LC | 39 | Forest, grassland, shrubland |
| 4 | Crested Goshawk | *Accipiter trivirgatus* (Temminck, 1824) | Acc.tri | LC | LC | 13 | Cropland, forest, grassland |
| 5 | Besra | *Accipiter virgatus* (Temminck, 1822) | Acc.vir | LC | LC | 7 | Forest, grassland |
| 6 | Cinereous Vulture | *Aegypius monachus* (Linnaeus, 1766) | Aeg.mon | NT | EN | 3 | Forest |
| 7 | Golden Eagle | *Aquila chrysaetos* (Linnaeus, 1758) | Aqu.chr | LC | VU | 2 | Grassland |
| 8 | Bonelli's Eagle | *Aquila fasciata* Vieillot, 1822 | Aqu.fas | LC | LC | 10 | Forest, grassland, shrubland |
| 9 | Steppe Eagle | *Aquila nipalensis* Hodgson, 1833 | Aqu.nip | EN | VU | 90 | Cropland, forest, grassland, shrubland, wetland |
| 10 | White-eyed Buzzard | *Butastur teesa* (Franklin, 1831) | But.tee | LC | LC | 1 | Forest |
| 11 | Common Buzzard | *Buteo buteo* (Linnaeus, 1758) | But.but | LC | LC | 4 | Forest |
| 12 | Upland Buzzard | *Buteo hemilasius* Temminck & Schlegel, 1844 | But.hem | LC | DD | 10 | Forest, grassland, shrubland |
| 13 | Himalayan Buzzard | *Buteo refectus* Portenko, 1929 | But.ref | LC | LC | 2 | Wetland |
| 14 | Short-toed Snake Eagle | *Circaetus gallicus* (Gmelin, 1788) | Cir.gal | LC | LC | 2 | Forest |
| 15 | Western Marsh Harrier | *Circus aeruginosus* (Linnaeus, 1758) | Cir.aer | LC | VU | 2 | Forest |
| 16 | Greater Spotted Eagle | *Clanga clanga* (Pallas, 1811) | Cla.cla | VU | VU | 5 | Shrubland |
| 17 | Indian Spotted Eagle | *Clanga hastata* (Lesson, 1831) | Cla.has | VU | VU | 1 | Forest |
| 18 | Bearded Vulture | *Gypaetus barbatus* (Linnaeus, 1758) | Gyp.bar | NT | VU | 17 | Forest, grassland, shrubland |
| 19 | White-rumped Vulture | *Gyps bengalensis* (Gmelin, 1788) | Gyp.ben | CR | CR | 12 | Forest, grassland, shrubland |
| 20 | Himalayan Griffon | *Gyps himalayensis* Hume, 1869 | Gyp.him | NT | VU | 9 | Cropland, grassland, shrubland |
| 21 | Slender-billed Vulture | *Gyps tenuirostris* Gray, 1844 | Gyp.ten | CR | CR | 5 | Forest |
| 22 | White-tailed Eagle | *Haliaeetus albicilla* (Linnaeus, 1758) | Hal.alb | LC | CR | 3 | Wetland, forest |
| 23 | Booted Eagle | *Hieraaetus pennatus* (Gmelin, 1788) | Hie.pen | LC | LC | 6 | Cropland, forest, shrubland |
| 24 | Gray-headed Fish Eagle | *Icthyophaga ichthyaetus* Horsfield, 1821 | Ict.ich | NT | CR | 3 | Forest |
| 25 | Black Eagle | *Ictinaetus malaiensis* (Temminck, 1822) | Ict.mal | LC | LC | 24 | Cropland, forest, grassland, shrubland, wetland |
| 26 | Black Kite | *Milvus migrans* (Boddaert, 1783) | Mil.mig | LC | LC | 52 | Cropland, forest, grassland, shrubland, wetland |
| 27 | Egyptian Vulture | *Neophron percnopterus* (Linnaeus, 1758) | Neo.per | EN | VU | 77 | Cropland, forest, grassland, shrubland, wetland |
| 28 | Changeable Hawk-eagle | *Nisaetus cirrhatus* (Gmelin, 1788) | Nis.cir | LC | LC | 1 | Shrubland |
| 29 | Mountain Hawk Eagle | *Nisaetus nipalensis* Hodgson, 1836 | Nis.nip | LC | LC | 3 | Cropland, forest |
| 30 | Osprey | *Pandion haliaetus* (Linnaeus, 1758) | Pan.hal | LC | LC | 15 | Forest |
| 31 | Oriental Honey-buzzard | *Pernis ptilorhynchus* (Temminck, 1821) | Per.pti | LC | LC | 11 | Forest |
| 32 | Red-headed Vulture | *Sarcogyps calvus* (Scopoli, 1786) | Sar.cal | CR | EN | 42 | Cropland, forest, grassland, shrubland |
| 33 | Crested Serpent Eagle | *Spilornis cheela* (Latham, 1790) | Spi.che | LC | LC | 29 | Cropland, forest, shrubland, wetland |
| **Order: Anseriformes** | | | | | | | |
| **Family: Anatidae** | | | | | | | |
| 34 | Northern Pintail | *Anas acuta* Linnaeus, 1758 | Ana.acu | LC | EN | 36 | Wetland |
| 35 | Mallard | *Anas platyrhynchos* Linnaeus, 1758 | Ana.pla | LC | LC | 32 | Wetland |
| 36 | Indian Spot-billed Duck | *Anas poecilorhyncha* Forster, 1781 | Ana.poe | LC | NT | 4 | Wetland |
| 37 | Garganey | *Anas querquedula* Linnaeus, 1758 | Ana.que | LC | VU | 12 | Wetland |
| 38 | Gadwall | *Anas strepera* Linnaeus, 1758 | Ana.str | LC | LC | 25 | Wetland |
| 39 | Greylag Goose | *Anser anser* (Linnaeus, 1758) | Ans.ans | LC | NT | 4 | Wetland |
| 40 | Bar-headed Goose | *Anser indicus* (Latham, 1790) | Ans.ind | LC | NT | 11 | Wetland |
| 41 | Common Pochard | *Aythya ferina* (Linnaeus, 1758) | Ayt.fer | VU | NT | 18 | Wetland |
| 42 | Tufted Duck | *Aythya fuligula* (Linnaeus, 1758) | Ayt.ful | LC | LC | 27 | Wetland |
| 43 | Ferruginous Duck | *Aythya nyroca* (Güldenstädt, 1770) | Ayt.nyr | LC | LC | 8 | Wetland |
| 44 | Common Goldeneye | *Bucephala clangula* (Linnaeus, 1758) | Buc.cla | LC | LC | 15 | Wetland |
| 45 | Lesser Whistling-duck | *Dendrocygna javanica* (Horsfield, 1821) | Den.jav | LC | LC | 44 | Wetland |
| 46 | Common Merganser | *Mergus merganser* Linnaeus, 1758 | Mer.mer | LC | LC | 7 | Wetland |
| 47 | Cotton Pygmy-goose | *Nettapus coromandelianus* (Gmelin, 1789) | Net.cor | LC | VU | 31 | Wetland |
| 48 | Northern Shoveler | *Spatula clypeata* (Linnaeus, 1758) | Spa.cly | LC | LC | 17 | Wetland |
| 49 | Ruddy Shelduck | *Tadorna ferruginea* Pallas, 1764 | Tad.fer | LC | LC | 26 | Wetland |
| **Order: Bucerotiformes** | | | | | | | |
| **Family: Upupidae** | | | | | | | |
| 50 | Common Hoopoe | *Upupa epops* Linnaeus, 1758 | Upu.epo | LC | LC | 54 | Forest, grassland, shrubland |
| **Order: Caprimulgiformes** | | | | | | | |
| **Family: Caprimulgiformes** | | | | | | | |
| 51 | Himalayan Swiftlet | *Aerodramus brevirostris* (Horsfield, 1840) | Aer.bre | LC | LC | 44 | Forest, grassland, shrubland |
| 52 | Common Swift | *Apus apus* (Linnaeus, 1758) | Apu.apu | LC | LC | 27 | Forest, grassland, shrubland |
| 53 | Nepal House Swift | *Apus nipalensis* (Hodgson, 1836) | Apu.nip | LC | LC | 147 | Forest, grassland, shrubland |
| 54 | Silver-backed Needletail | *Hirundapus cochinchinensis* (Oustalet, 1878) | Hir.coc | LC | LC | 4 | Forest |
| 55 | Alpine Swift | *Tachymarptis melba* (Linnaeus, 1758) | Tac.mel | LC | LC | 98 | Cropland, forest, grassland, shrubland, wetland |
| **Family: Caprimulgidae** | | | | | | | |
| 56 | Savanna Nightjar | *Caprimulgus affinis* Horsfield, 1821 | Cap.aff | LC | LC | 8 | Forest, wetland |
| 57 | Gray Nightjar | *Caprimulgus indicus* (Latham), 1790 | Cap.ind | LC | LC | 4 | Forest, grassland |
| **Family: Hemiprocnidae** | | | | | | | |
| 58 | Crested Treeswift | *Hemiprocne coronata* (Tickell, 1833) | Hem.cor | LC | LC | 3 | Forest |
| **Order: Charadriiformes** | | | | | | | |
| **Family: Charadriidae** | | | | | | | |
| 59 | Kentish Plover | *Charadrius alexandrinus* Linnaeus, 1758 | Cha.ale | LC | LC | 5 | Forest |
| 60 | Little Ringed Plover | *Charadrius dubius* Scopoli, 1786 | Cha.dub | LC | LC | 35 | Forest, wetland |
| 61 | Long-billed Plover | *Charadrius placidus* Gray & Gray, 1863 | Cha.pla | LC | LC | 1 | Forest |
| 62 | Pacific Golden Plover | *Pluvialis fulva* (Gmelin, 1789) | Plu.ful | LC | NT | 2 | Forest |
| 63 | Gray-headed Lapwing | *Vanellus cinereus* (Blyth, 1842) | Van.cin | LC | LC | 6 | Wetland, forest |
| 64 | River Lapwing | *Vanellus duvaucelii* (Lesson, 1826) | Van.duv | NT | NT | 11 | Forest, grassland |
| 65 | Red-wattled Lapwing | *Vanellus indicus* (Boddaert, 1783) | Van.ind | LC | LC | 112 | Forest, grassland, shrubland, wetland |
| **Family: Ibidorhynchidae** | | | | | | | |
| 66 | Ibisbill | *Ibidorhyncha struthersii* Vigors, 1832 | Ibi.str | LC | EN | 3 | Forest |
| **Family: Jacanidae** | | | | | | | |
| 67 | Pheasant-tailed Jacana | *Hydrophasianus chirurgus* (Scopoli, 1786) | Hyd.chi | LC | VU | 6 | Wetland |
| 68 | Bronze-winged Jacana | *Metopidius indicus* (Latham, 1790) | Met.ind | LC | LC | 4 | Forest |
| **Family: Rostratulidae** | | | | | | | |
| 69 | Greater Painted-snipe | *Rostratula benghalensis* (Linnaeus, 1758) | Ros.ben | LC | LC | 3 | Wetland |
| **Family: Scolopacidae** | | | | | | | |
| 70 | Common Sandpiper | *Actitis hypoleucos* Linnaeus, 1758 | Act.hyp | LC | LC | 14 | Wetland, shrubland |
| 71 | Little Stint | *Calidris minuta* (Leisler, 1812) | Cal.min | LC | LC | 3 | Grassland |
| 72 | Common Snipe | *Gallinago gallinago* (Linnaeus, 1758) | Gal.gal1 | LC | LC | 31 | Forest, shrubland, wetland |
| 73 | Pin-tailed Snipe | *Gallinago stenura* (Bonaparte, 1830) | Gal.ste | LC | LC | 4 | Forest |
| 74 | Eurasian Woodcock | *Scolopax rusticola* Linnaeus, 1758 | Sco.rus | LC | LC | 10 | Forest |
| 75 | Wood Sandpiper | *Tringa glareola* Linnaeus, 1758 | Tri.gla | LC | LC | 5 | Wetland |
| 76 | Common Greenshank | *Tringa nebularia* (Gunnerus, 1767) | Tri.neb | LC | LC | 2 | Wetland |
| 77 | Green Sandpiper | *Tringa ochropus* Linnaeus, 1758 | Tri.och | LC | LC | 5 | Wetland |
| 78 | Common Redshank | *Tringa totanus* (Linnaeus, 1758) | Tri.tot | LC | LC | 5 | Forest |
| **Order: Ciconiiformes** | | | | | | | |
| **Family: Ardeidae** | | | | | | | |
| 79 | Intermediate Egret | *Ardea intermedia* Wagler, 1829 | Ard.int | LC | LC | 55 | Cropland, forest, grassland, shrubland, wetland |
| 80 | Indian Pond Heron | *Ardeola grayii* (Sykes, 1832) | Ard.gra | LC | LC | 46 | Cropland, forest, grassland, shrubland, wetland |
| 81 | Cattle Egret | *Bubulcus ibis* (Linnaeus, 1758) | Bub.ibi | LC | LC | 130 | Cropland, forest, grassland, shrubland, wetland |
| 82 | Great Egret | *Casmerodius albus* (Linnaeus, 1758) | Cas.alb | LC | LC | 31 | Cropland, forest, grassland, shrubland, wetland |
| 83 | Little Egret | *Egretta garzetta* (Linnaeus, 1766) | Egr.gar | LC | LC | 24 | Forest, shrubland, wetland |
| **Family: Ciconiidae** | | | | | | | |
| 84 | Asian Openbill | *Anastomus oscitans* (Boddaert, 1783) | Ana.osc | LC | VU | 4 | Wetland |
| 85 | Asian Woollyneck | *Ciconia episcopus* (Boddaert, 1783) | Cic.epi | VU | NT | 7 | Wetland |
| 86 | Black Stork | *Ciconia nigra* (Linnaeus, 1758) | Cic.nig | LC | VU | 6 | Wetland |
| 87 | Lesser Adjutant | *Leptoptilos javanicus* (Horsfield, 1821) | Lep.jav | VU | VU | 2 | Wetland |
| **Order: Columbiformes** | | | | | | | |
| **Family: Columbidae** | | | | | | | |
| 88 | Emerald Dove | *Chalcophaps indica* (Linnaeus, 1758) | Cha.ind | LC | LC | 34 | Cropland, forest, grassland, shrubland, wetland |
| 89 | Speckled Wood Pigeon | *Columba hodgsonii* Vigors, 1832 | Col.hod | LC | LC | 7 | Shrubland |
| 90 | Rock Dove | *Columba livia* Gmelin, 1789 | Col.liv | LC | LC | 84 | Cropland, forest, grassland, shrubland |
| 91 | Common Wood Pigeon | *Columba palumbus* Linnaeus, 1758 | Col.pal | LC | LC | 34 | Cropland, forest, grassland, shrubland |
| 92 | Ashy Wood Pigeon | *Columba pulchricolli*s Blyth, 1846 | Col.pul | LC | LC | 3 | Forest |
| 93 | Barred Cuckoo Dove | *Macropygia unchall*(Wagler, 1827) | Mac.unc | LC | VU | 2 | Grassland |
| 94 | Spotted Dove | *Spilopelia suratensis* (Gmelin, 1789) | Spi.sur | LC | LC | 87 | Cropland, forest, grassland, shrubland, wetland |
| 95 | Eurasian Collared Dove | *Streptopelia decaocto* Frivaldszky, 1838 | Str.dec | LC | LC | 11 | Grassland, shrubland |
| 96 | Oriental Turtle Dove | *Streptopelia orientalis* (Latham, 1790) | Str.ori | LC | LC | 42 | Cropland, forest, grassland, shrubland, wetland |
| 97 | Red Turtle-dove | *Streptopelia tranquebarica* (Hermann, 1804) | Str.tra | LC | LC | 4 | Grassland |
| 98 | Orange-breasted Green Pigeon | *Treron bicinctus* (Jerdon, 1840) | Tre.bic | LC | LC | 2 | Wetland |
| 99 | Yellow-footed Green Pigeon | *Treron phoenicopterus* (Latham, 1790) | Tre.pho | LC | LC | 30 | Cropland, forest, grassland, shrubland, wetland |
| 100 | Wedge-tailed Green Pigeon | *Treron sphenurus* (Vigors, 1832) | Tre.sph | LC | LC | 19 | Forest, shrubland |
| 101 | Grey-bellied Cuckoo | *Cacomantis passerinus* (Vahl, 1797) | Cac.pas | LC | LC | 1 | Cropland |
| **Order: Coraciiformes** | | | | | | | |
| **Family:Alcedinidae** | | | | | | | |
| 102 | Common Kingfisher | *Alcedo atthis* (Linnaeus, 1758) | Alc.att | LC | LC | 35 | Cropland, forest, grassland, shrubland |
| 103 | Pied Kingfisher | *Ceryle rudis* (Linnaeus, 1758) | Cer.rud | LC | LC | 9 | Shrubland |
| 104 | White-throated Kingfisher | *Halcyon gularis* (Kuhl, 1820) | Hal.gul | LC | LC | 48 | Forest, grassland, shrubland, wetland |
| 105 | Crested Kingfisher | *Megaceryle lugubris* (Temminck, 1834) | Meg.lug | LC | LC | 16 | Forest, grassland, shrubland, wetland |
| **Family: Coraciidae** | | | | | | | |
| 106 | Indian Roller | *Coracias benghalensis* (Linnaeus, 1758) | Cor.ben | LC | LC | 47 | Cropland, forest, grassland, shrubland |
| 107 | Oriental Dollarbird | *Eurystomus orientalis* (Linnaeus, 1766) | Eur.ori | LC | LC | 11 | Forest, grassland, shrubland |
| **Family: Meropidae** | | | | | | | |
| 108 | Chestnut-headed Bee-eater | *Merops leschenaulti* Vieillot, 1817 | Mer.les | LC | LC | 2 | Shrubland |
| 109 | Blue-tailed Bee-eater | *Merops philippinus* Linnaeus, 1766 | Mer.phi | LC | LC | 4 | Cropland, shrubland |
| 110 | Green Bee-eater | *Merops viridissimus* Swainson, 1837 | Mer.vir | LC | LC | 14 | Forest, grassland, cropland |
| 111 | Blue-bearded Bee-eater | *Nyctyornis athertoni* (Jardine & Selby, 1830) | Nyc.ath | LC | LC | 9 | Cropland, forest, wetland |
| **Order: Cuculiformes** | | | | | | | |
| **Family: Cuculidae** | | | | | | | |
| 112 | Banded Bay Cuckoo | *Cacomantis sonneratii* (Latham, 1790) | Cac.son | LC | LC | 1 | Shrubland |
| 113 | Lesser Coucal | *Centropus bengalensis* (Gmelin, 1788) | Cen.ben | LC | LC | 15 | Cropland, forest, grassland |
| 114 | Greater Coucal | *Centropus sinensis* (Stephens, 1815) | Cen.sin | LC | LC | 29 | Cropland, forest, grassland, shrubland, wetland |
| 115 | Common Cuckoo | *Cuculus canorus* Linnaeus, 1758 | Cuc.can | LC | LC | 55 | Cropland, forest, grassland,shrubland, wetland |
| 116 | Lesser Cuckoo | *Cuculus poliocephalus* Latham, 1790 | Cuc.pol | LC | LC | 5 | Shrubland, forest |
| 117 | Western Koel | *Eudynamys scolopaceus* (Linnaeus, 1758) | Eud.sco | LC | LC | 192 | Cropland, forest, grassland, shrubland, wetland |
| 118 | Large Hawk Cuckoo | *Hierococcyx sparverioides* (Vigors, 1831) | Hie.spa | LC | LC | 28 | Cropland, forest, grassland, shrubland |
| 119 | Common Hawk-cuckoo | *Hierococcyx varius* (Vahl, 1797) | Hie.var | LC | LC | 2 | Cropland |
| 120 | Green-billed Malkoha | *Phaenicophaeus tristis* (Lesson, 1830) | Pha.tri | LC | LC | 10 | Shrubland, cropland, forest |
| 121 | Square-tailed Drongo-cuckoo | *Surniculus lugubris* (Horsfield, 1821) | Sur.lug | LC | LC | 49 | Cropland, forest, grassland, shrubland, wetland |
| **Falconiformes** | | | | | | | |
| **Falconidae** | | | | | | | |
| 122 | Amur Falcon | *Falco amurensis* Radde, 1863 | Fal.amu | LC | LC | 2 | Forest |
| 123 | Saker Falcon | *Falco cherrug* Gray, 1834 | Fal.che | EN | EN | 8 | Cropland, forest |
| 124 | Lesser Kestrel | *Falco naumanni* Fleischer, 1818 | Fal.nau | LC | NT | 2 | Forest |
| 125 | Peregrine Falcon | *Falco peregrinus* Tunstall, 1771 | Fal.per | LC | LC | 12 | Forest, grassland |
| 126 | Common Kestrel | *Falco tinnunculus* Linnaeus, 1758 | Fal.tin | LC | LC | 48 | Cropland, forest, grassland, shrubland, wetland |
| 127 | Collared Falconet | *Microhierax caerulescens* (Linnaeus, 1758) | Mic.cae | LC | NT | 6 | Forest |
| **Order: Galliformes** | | | | | | | |
| **Family: Phasianidae** | | | | | | | |
| 128 | Black Francolin | *Francolinus francolinus* (Linnaeus, 1766) | Fra.fra | LC | LC | 25 | Cropland, forest, grassland, shrubland, wetland |
| 129 | Red Junglefowl | *Gallus gallus* (Linnaeus, 1758) | Gal.gal | LC | LC | 7 | Forest, shrubland |
| 130 | Blood Pheasant | *Ithaginis cruentus* (Hardwicke, 1821) | Ith.cru | LC | LC | 3 | Forest |
| 131 | Kalij Pheasant | *Lophura leucomelanos* (Latham, 1790) | Lop.leu | LC | LC | 31 | Cropland, forest, grassland, shrubland, wetland |
| 132 | Indian Peafowl | *Pavo cristatus* Linnaeus, 1758 | Pav.cri | LC | NT | 8 | Forest, Grassland |
| **Order: Gruiformes** | | | | | | | |
| **Family: Hirundinidae** | | | | | | | |
| 133 | Asian House Martin | *Delichon dasypus* (Bonaparte, 1850) | Del.das | LC | LC | 19 | Forest, grassland, shrubland |
| **Family: Rallidae** | | | | | | | |
| 134 | White-breasted Waterhen | *Amaurornis phoenicurus* (Pennant, 1769) | Ama.pho | LC | LC | 42 | Shrubland, wetland |
| 135 | Common Coot | *Fulica atra* Linnaeus, 1758 | Ful.atr | LC | LC | 34 | Wetland |
| 136 | Common Moorhen | *Gallinula chloropus* (Linnaeus, 1758) | Gal.chl | LC | LC | 42 | Wetland |
| 137 | Purple Swamphen | *Porphyrio porphyrio* (Linnaeus, 1758) | Por.por | LC | LC | 66 | Wetland |
| 138 | Brown Crake | *Zapornia akool* (Sykes, 1832) | Zap.ako | LC | LC | 3 | Forest |
| 139 | Ruddy-breasted Crake | *Zapornia fusca* (Linnaeus, 1766) | Zap.fus | LC | LC | 4 | Wetland |
| **Order: Passeriformes** | | | | | | | |
| **Family: Acrocephalidae** | | | | | | | |
| 140 | Blyth's Reed Warbler | *Acrocephalus dumetorum* Blyth, 1849 | Acr.dum | LC | LC | 50 | Cropland, forest, grassland, shrubland |
| **Family: Aegithalidae** | | | | | | | |
| 141 | Black-throated Tit | *Aegithalos concinnus* (Gould, 1855) | Aeg.con | LC | LC | 28 | Cropland, forest, grassland, shrubland |
| 142 | Rufous-fronted Tit | *Aegithalos iouschistos* (Blyth, 1845) | Aeg.iou | LC | LC | 3 | Shrubland |
| 143 | Common Iora | *Aegithina tiphia* (Linnaeus, 1758) | Aeg.tip | LC | LC | 2 | Shrubland |
| **Family: Alaudidae** | | | | | | | |
| 144 | Oriental Skylark | *Alauda gulgula* Franklin, 1831 | Ala.gul | LC | LC | 7 | Forest, wetland |
| 145 | Sand Lark | *Alaudala raytal* (Blyth, 1844) | Ala.ray | LC | LC | 2 | Forest, wetland |
| 146 | Hume's Short-toed Lark | *Calandrella acutirostris* Hume, 1873 | Cal.acu | LC | LC | 4 | Cropland |
| 147 | Greater Short-toed Lark | *Calandrella brachydactyla* (Leisler, 1814) | Cal.bra | LC | LC | 11 | Cropland, forest, grassland, shrubland |
| 148 | Bengal Bushlark | *Mirafra assamica* Horsfield, 1840 | Mir.ass | LC | LC | 13 | Forest,shrubland,wetland |
| **Family: Alcippeidae** | | | | | | | |
| 149 | Nepal Fulvetta | *Alcippe nipalensis* (Hodgson, 1837) | Alc.nip | LC | LC | 20 | Cropland, forest, grassland |
| **Family: Artamidae** | | | | | | | |
| 150 | Ashy Woodswallow | *Artamus fuscus* Vieillot, 1817 | Art.fus | LC | LC | 50 | Cropland, forest, shrubland, wetland |
| **Family: Campephagidae** | | | | | | | |
| 151 | Large Cuckooshrike | *Coracina javensis* (Horsfield, 1821) | Cor.jav | LC | LC | 18 | Cropland, forest, shrubland, wetland |
| 152 | Black-winged Cuckooshrike | *Coracina melaschistos* (Hodgson, 1836) | Cor.mel | LC | LC | 34 | Cropland, forest, grassland, shrubland, wetland |
| 153 | Indian Cuckoo | *Cuculus micropterus* Gould, 1837 | Cuc.mic | LC | LC | 75 | Cropland, forest, grassland, shrubland, wetland |
| 154 | Short-billed Minivet | *Pericrocotus brevirostris* (Vigors, 1831) | Per.bre | LC | LC | 34 | Cropland, forest, grassland, shrubland, wetland |
| 155 | Small Minivet | *Pericrocotus cinnamomeus* Linnaeus, 1766 | Per.cin | LC | LC | 4 | Forest |
| 156 | Long-tailed Minivet | *Pericrocotus ethologus* Bangs & Phillips, 1914 | Per.eth | LC | LC | 53 | Cropland, forest, grassland, shrubland |
| 157 | Scarlet Minivet | *Pericrocotus flammeus* (Forster, 1781) | Per.fla | LC | LC | 93 | Cropland, forest, grassland, shrubland, wetland |
| 158 | Gray-chinned Minivet | *Pericrocotus solaris* Blyth, 1846 | Per.sol | LC | LC | 2 | Forest |
| **Family: Certhiidae** | | | | | | | |
| 159 | Sikkim Treecreeper | *Certhia discolor* Blyth, 1845 | Cer.dis | LC | LC | 6 | Forest, shrubland, wetland |
| 160 | Bar-tailed Treecreeper | *Certhia himalayana* Vigors, 1832 | Cer.him | LC | LC | 3 | Forest |
| 161 | Hodgson's Treecreeper | *Certhia hodgsoni* W. E. Brooks, 1871 | Cer.hod | LC | LC | 5 | Shrubland |
| 162 | Rusty-flanked Treecreeper | *Certhia nipalensis* Blyth, 1855 | Cer.nip | LC | LC | 14 | Forest, shrubland, grassland |
| **F amily: Chloropseidae** | | | | | | | |
| 163 | Golden-fronted Leaf Bird | *Chloropsis aurifrons* (Temminck, 1829) | Chl.aur | LC | LC | 2 | Cropland |
| 164 | Orange-bellied Leafbird | *Chloropsis hardwickii* (Jardine & Selby, 1830) | Chl.har | LC | LC | 34 | Cropland, forest, grassland, shrubland, wetland |
| **Family: Cinclidae** | | | | | | | |
| 165 | Brown Dipper | *Cinclus pallasii* Temminck, 1820 | Cin.pal | LC | LC | 8 | Forest, grassland, wetland |
| **Family: Cisticolidae** | | | | | | | |
| 166 | Zitting Cisticola | *Cisticola juncidis* (Rafinesque, 1810) | Cis.jun | LC | LC | 12 | Forest, grassland, Shrubland |
| 167 | Common Tailorbird | *Orthotomus sutorius* (Pennant, 1769) | Ort.sut | LC | LC | 25 | Cropland, forest, grassland, shrubland, wetland |
| 168 | Gray-crowned Prinia | *Prinia cinereocapilla* Hodgson, 1854 | Pri.cin | VU | CR | 3 | Grassland |
| 169 | Striated Prinia | *Prinia crinigera* Hodgson, 1836 | Pri.cri | LC | LC | 62 | Cropland, forest, grassland, shrubland, wetland |
| 170 | Yellow-bellied Prinia | *Prinia flaviventris* (Delessert, 1840) | Pri.fla | LC | NT | 4 | Wetland |
| 171 | Gray-breasted Prinia | *Prinia hodgsonii* Blyth, 1844 | Pri.hod | LC | LC | 30 | Forest, grassland, shrubland, wetland |
| 172 | Plain Prinia | *Prinia inornata* Sykes, 1832 | Pri.ino | LC | LC | 4 | Grassland |
| 173 | Ashy Prinia | *Prinia socialis* Sykes, 1832 | Pri.soc | LC | LC | 4 | Cropland |
| **Family: Corvidae** | | | | | | | |
| 174 | Common Green Magpie | *Cissa chinensis* (Boddaert, 1783) | Cis.chi | LC | LC | 69 | Cropland, forest, grassland, shrubland, wetland |
| 175 | Common Raven | *Corvus corax* Linnaeus, 1758 | Cor.cor | LC | LC | 34 | Forest, grassland, shrubland |
| 176 | Large-billed Crow | *Corvus macrorhynchos* Wagler, 1827 | Cor.mac | LC | LC | 35 | Cropland, forest, grassland, shrubland |
| 177 | House Crow | *Corvus splendens* Vieillot, 1817 | Cor.spl | LC | LC | 61 | Forest, grassland, shrubland, wetland |
| 178 | Grey Treepie | *Dendrocitta formosae* Swinhoe, 1863 | Den.for | LC | LC | 68 | Cropland, forest, grassland, shrubland, wetland |
| 179 | Rufous Treepie | *Dendrocitta vagabunda* (Latham, 1790) | Den.vag | LC | LC | 27 | Forest, shrubland |
| 180 | Eurasian Jay | *Garrulus glandarius* (Linnaeus, 1758) | Gar.gla | LC | LC | 14 | Cropland, forest, grassland, shrubland |
| 181 | Black-headed Jay | *Garrulus lanceolatus* Vigors, 1831 | Gar.lan | LC | LC | 3 | Forest |
| 182 | Northern Nutcracker | *Nucifraga caryocatactes* (Linnaeus, 1758) | Nuc.car | LC | LC | 3 | Forest |
| 183 | Alpine Chough | *Pyrrhocorax graculus* Linnaeus, 1766 | Pyr.gra | LC | LC | 23 | Forest, grassland, shrubland, wetland |
| 184 | Red-billed Chough | *Pyrrhocorax pyrrhocorax* Linnaeus, 1758 | Pyr.pyr | LC | LC | 17 | grassland, shrubland |
| 185 | Red-billed Blue Magpie | *Urocissa erythroryncha* (Boddaert, 1783) | Uro.ery | LC | LC | 116 | Cropland, forest, grassland, shrubland |
| 186 | Yellow-billed Blue Magpie | *Urocissa flavirostris* (Blyth, 1846) | Uro.fla | LC | LC | 28 | Forest, shrubland |
| **Family: Dicaeidae** | | | | | | | |
| 187 | Thick-billed Flowerpecker | *Dicaeum agile* (Tickell, 1833) | Dic.agi | LC | LC | 6 | Forest |
| 188 | Fire-breasted Flowerpecker | *Dicaeum ignipectus* (Blyth, 1843) | Dic.ign | LC | LC | 10 | Cropland, forest, grassland |
| 189 | Yellow-bellied Flowerpecker | *Dicaeum melanoxanthum* Blyth, 1843 | Dic.mel | LC | LC | 3 | Forest |
| 190 | Bronzed Drongo | *Dicrurus aeneus* Vieillot, 1817 | Dic.aen | LC | LC | 51 | Cropland, forest, grassland, shrubland, wetland |
| 191 | Crow-billed Drongo | *Dicrurus annectans* (Hodgson, 1836) | Dic.ann | LC | LC | 3 | Forest |
| 192 | White-bellied Drongo | *Dicrurus caerulescens* (Linnaeus, 1758) | Dic.cae | LC | LC | 4 | Shrubland |
| 193 | Spangled Drongo | *Dicrurus hottentottus* (Linnaeus, 1766) | Dic.hot | LC | LC | 62 | Cropland, forest, grassland, shrubland |
| 194 | Ashy Drongo | *Dicrurus leucophaeus* Vieillot, 1817 | Dic.leu | LC | LC | 38 | Forest, grassland, shrubland |
| 195 | Black Drongo | *Dicrurus macrocercus* Vieillot, 1817 | Dic.mac | LC | LC | 29 | Cropland, forest, grassland, shrubland |
| 196 | Greater Racquet-tailed Drongo | *Dicrurus paradiseus* (Linnaeus, 1766) | Dic.par | LC | LC | 4 | Shrubland |
| 197 | Lesser Racket-tailed Drongo | *Dicrurus remifer* (Temminck, 1823) | Dic.rem | LC | LC | 7 | Forest, shrubland |
| **Family: Emberizidae** | | | | | | | |
| 198 | Rock Bunting | *Emberiza cia* Linnaeus, 1766 | Emb.cia | LC | LC | 11 | Forest,grassland, shrubland |
| 199 | Chestnut-eared Bunting | *Emberiza fucata* Pallas, 1766 | Emb.fuc | LC | LC | 9 | Forest, shrubland, wetland |
| 200 | Crested Bunting | *Emberiza lathami* Gray, 1831 | Emb.lat | LC | LC | 59 | Cropland, forest, grassland, shrubland, wetland |
| 201 | Black-headed Bunting | *Emberiza melanocephala* | Emb.mel | LC | VU | 2 | Cropland |
| 202 | Little Bunting | *Emberiza pusilla* Pallas, 1776 | Emb.pus | LC | VU | 16 | Forest, grassland |
| **Family: Estrildidae** | | | | | | | |
| 203 | Rufous-throated partridge | *Arborophila rufogularis* (Blyth, 1850) | Arb.ruf | LC | NT | 2 | Shrubland |
| 204 | Chestnut Munia | *Lonchura atricapilla* (Vieillot, 1807) | Lon.atr | LC | LC | 3 | Grassland |
| 205 | Tricoloured Munia | *Lonchura malacca* (Linnaeus, 1766) | Lon.mal | LC | LC | 2 | Shrubland |
| 206 | Scaly-breasted Munia | *Lonchura punctulata* (Linnaeus, 1758) | Lon.pun | LC | LC | 134 | Cropland, forest, grassland, shrubland, wetland |
| 207 | White-rumped Munia | *Lonchura striata* (Linnaus, 1766) | Lon.str | LC | LC | 15 | Forest, grassland, shrubland |
| **Family: Eurylaimidae** | | | | | | | |
| 208 | Long-tailed Broadbill | *Psarisomus dalhousiae* (Jameson, 1835) | Psa.dal | LC | LC | 4 | Shrubland, grassland |
| **Family: Fringillidae** | | | | | | | |
| 209 | Blanford's Rosefinch | *Agraphospiza rubescens* (Blanford, 1872) | Agr.rub | LC | LC | 2 | Shrubland |
| 210 | European Goldfinch | *Carduelis carduelis* (Linnaeus 1758) | Car.car | LC | LC | 10 | Cropland, forest, shrubland |
| 211 | Yellow-breasted Greenfinch | *Carduelis spinoides* (Vigors, 1831) | Car.spi | LC | LC | 34 | Cropland, forest, grassland, shrubland |
| 212 | Common Rosefinch | *Carpodacus erythrinus* (Pallas, 1770) | Car.ery | LC | LC | 73 | Forest, grassland, shrubland, wetland |
| 213 | Beautiful Rosefinch | *Carpodacus pulcherrimus* (F. Moore 1856) | Car.pul | LC | LC | 35 | Forest, grassland, shrubland |
| 214 | Red-fronted Rosefinch | *Carpodacus puniceus* (Blyth, 1845) | Car.pun | LC | LC | 14 | Forest, grassland, shrubland |
| 215 | Pink-browed Rosefinch | *Carpodacus rodochroa* (Vigors, 1831) | Car.rod | LC | LC | 8 | Forest, shrubland |
| 216 | Spot-winged Rosefinch | *Carpodacus rodopeplus* (Vigors 1831) | Car.rod1 | LC | LC | 7 | Shrubland |
| 217 | Great Rosefinch | *Carpodacus rubicilla* (Güldenstädt, 1775) | Car.rub | LC | LC | 5 | Shrubland |
| 218 | Scarlet Finch | *Carpodacus sipahi* (Hodgson, 1836) | Car.sip | LC | NT | 36 | Forest, grassland, shrubland, wetland |
| 219 | Crimson-browed Finch | *Carpodacus subhimachalus* (Hodgson, 1836) | Car.sub | LC | LC | 7 | Wetland, shrubland |
| 220 | Himalayan White-browed Rosefinch | *Carpodacus thura* Bonaparte & Schlegel, 1850 | Car.thu | LC | LC | 6 | Forest, grassland |
| 221 | Brambling | *Fringilla montifringilla* Linnaeus, 1758 | Fri.mon | LC | LC | 2 | Grassland |
| 222 | Plain Mountain Finch | *Leucosticte nemoricola* (Hodgson, 1836) | Leu.nem | LC | LC | 2 | Shrubland |
| 223 | Collared Grosbeak | *Mycerobas affinis* (Blyth, 1855) | Myc.aff | LC | LC | 6 | Forest, shrubland |
| 224 | White-Winged Grosbeak | *Mycerobas carnipes* (Hodgson, 1836) | Myc.car | LC | LC | 20 | Forest, grassland, shrubland, wetland |
| 225 | Dark-breasted Rosefinch | *Procarduelis nipalensis* (Hodgson, 1836) | Pro.nip | LC | LC | 14 | Grassland, shrubland, wetland |
| 226 | Red-headed Bullfinch | *Pyrrhula erythrocephala* Vigors, 1832 | Pyr.ery | LC | LC | 29 | Forest, grassland, shrubland |
| **Family: Hirundinidae** | | | | | | | |
| 227 | Red-rumped Swallow | *Cecropis daurica* Linnaeus, 1771 | Cec.dau | LC | LC | 112 | Cropland, forest, grassland, shrubland, wetland |
| 228 | Nepal House Martin | *Delichon nipalense* Horsfield & Moore, 1854 | Del.nip | LC | LC | 75 | Forest, grassland, shrubland, wetland |
| 229 | Eurasian Crag Martin | *Hirundo rupestris* (Scopoli 1769) | Hir.rup | LC | LC | 5 | Cropland, grassland |
| 230 | Barn Swallow | *Hirundo rustica* Linnaeus, 1758 | Hir.rus | LC | LC | 34 | Cropland, forest, grassland, shrubland |
| 231 | Plain Martin | *Riparia paludicola* (Vieillot, 1817) | Rip.pal | LC | LC | 40 | Cropland, forest, grassland, shrubland, wetland |
| **Family: Laniidae** | | | | | | | |
| 232 | Brown Shrike | *Lanius cristatus Linnaeus*, 1758 | Lan.cri | LC | LC | 43 | Forest, grassland, shrubland, wetland |
| 233 | Long-tailed Shrike | *Lanius schach Linnaeus*, 1758 | Lan.sch | LC | LC | 70 | Cropland, forest, grassland, shrubland, wetland |
| 234 | Gray-backed Shrike | *Lanius tephronotus* (Vigors, 1831) | Lan.tep | LC | LC | 23 | Forest, grassland, shrubland, wetland |
| **Leiotrichidae** | | | | | | | |
| 235 | Hoary-throated Barwing | *Sibia nipalensis* (Hodgson, 1836) | Act.nip | LC | LC | 2 | Forest |
| 236 | Striated Babbler | *Argya earlei* (Blyth, 1844) | Arg.ear | LC | LC | 13 | Forest, cropland, grassland |
| 237 | Jungle Babbler | *Argya striata* (Dumont, 1823) | Arg.str | LC | LC | 67 | Forest, grassland, shrubland |
| 238 | White-throated Laughingthrush | *Garrulax albogularis* (Gould, 1836) | Gar.alb | LC | LC | 63 | Cropland, forest, grassland, shrubland, wetland |
| 239 | White-crested Laughingthrush | *Garrulax leucolophus* (Hardwicke, 1815) | Gar.leu | LC | LC | 41 | Forest, grassland, wetland |
| 240 | Striated Laughingthrush | *Grammatoptila striata* (Vigors, 1831) | Gra.str | LC | LC | 3 | Cropland |
| 241 | Rufous Sibia | *Heterophasia capistrata* (Vigors, 1831) | Het.cap | LC | LC | 15 | Cropland, forest |
| 242 | Red-billed Leiothrix | *Leiothrix lutea* (Scopoli, 1786) | Lei.lut | LC | LC | 21 | Cropland, forest, grassland, shrubland |
| 243 | Red-tailed Minla | *Minla ignotincta* Hodgson, 1837 | Min.ign | LC | LC | 3 | Min.ign |
| 244 | Blue-winged Minla | *Siva cyanouroptera* Hodgson, 1837 | Siv.cya | LC | LC | 2 | Shrubland |
| 245 | Spiny Babbler | *Turdoides nipalensis* (Hodgson, 1836) | Tur.nip | LC | LC | 18 | Cropland, forest, grassland, shrubalnd |
| **Family: Motacillidae** | | | | | | | |
| 246 | Tawny Pipit | *Anthus campestris* (Linnaeus, 1758) | Ant.cam | LC | LC | 2 | Wetland |
| 247 | Red-throated Pipit | *Anthus cervinus* (Pallas, 1811) | Ant.cer | LC | LC | 8 | Forest, grassland |
| 248 | Blyth's Pipit | *Anthus godlewskii* (Taczanowski 1876) | Ant.god | LC | LC | 6 | Forest, cropland |
| 249 | Olive-backed Pipit | *Anthus hodgsoni* Richmond, 1907 | Ant.hod | LC | LC | 41 | Forest, grassland, shrubland |
| 250 | Richard's Pipit | *Anthus richardi* Vieillot, 1818 | Ant.ric | LC | LC | 18 | Cropland, forest, grassland, shrubland, wetland |
| 251 | Rosy Pipit | *Anthus roseatus* Blyth, 1847 | Ant.ros | LC | LC | 81 | Cropland, forest, grassland, shrubland, wetland |
| 252 | Paddyfield Pipit | *Anthus rufulus* Vieillot, 1818 | Ant.ruf | LC | LC | 33 | Cropland, forest, grassland, shrubland, wetland |
| 253 | Upland Pipit | *Anthus sylvanus* (Blyth, 1845) | Ant.syl | LC | LC | 3 | Forest |
| 254 | Tree Pipit | *Anthus trivialis* (Linnaeus, 1758) | Ant.tri | LC | LC | 3 | Shrubland |
| 255 | Forest Wagtail | *Dendronanthus indicus* (Gmelin, 1789) | Den.ind | LC | LC | 3 | Grassland |
| 256 | White Wagtail | *Motacilla alba* Linnaeus, 1758 | Mot.alb | LC | LC | 85 | Cropland, forest, grassland, shrubland, wetland |
| 257 | Gray Wagtail | *Motacilla cinerea* Tunstall, 1771 | Mot.cin | LC | LC | 19 | Forest, shrubland, wetland |
| 258 | Citrine Wagtail | *Motacilla citreola* Pallas, 1776 | Mot.cit | LC | LC | 13 | Forest, grassland, wetland |
| 259 | Yellow Wagtail | *Motacilla flava* Linnaeus, 1758 | Mot.fla | LC | LC | 28 | Cropland, forest, grassland, shrubland, wetland |
| 260 | White-browed Wagtail | *Motacilla maderaspatensis* Gmelin, 1789 | Mot.mad | LC | LC | 58 | Cropland, forest, grassland, shrubland, wetland |
| **Family: Muscicapidae** | | | | | | | |
| 261 | Siberian Rubythroat | *Calliope calliope* (Pallas, 1776) | Cal.cal | LC | LC | 3 | Forest |
| 262 | Himalayan Rubythroat | *Calliope pectoralis* (Gould, 1837) | Cal.pec | LC | LC | 13 | Forest, shrubland |
| 263 | Indian Robin | *Copsychus fulicatus* (Linnaeus, 1766) | Cop.ful | LC | LC | 1 | Forest |
| 264 | Oriental Magpie Robin | *Copsychus saularis* (Linnaeus, 1758) | Cop.sau | LC | LC | 76 | Cropland, forest, grassland, shrubland, wetland |
| 265 | Pale-chinned Flycatcher | *Cyornis poliogenys* Brooks, 1879 | Cyo.pol | LC | LC | 2 | Shrubland |
| 266 | Blue-throated Blue Flycatcher | *Cyornis rubeculoides* (Vigors, 1831) | Cyo.rub | LC | LC | 4 | Cropland, shrubland |
| 267 | Pale Blue Flycatcher | *Cyornis unicolor* Blyth, 1843 | Cyo.uni | LC | LC | 39 | Forest, grassland, shrubland |
| 268 | Black-backed Forktail | *Enicurus immaculatus* (Hodgson, 1836) | Eni.imm | LC | LC | 11 | Forest, grassland, shrubland, wetland |
| 269 | Spotted Forktail | *Enicurus maculatus* Vigors, 1831 | Eni.mac | LC | LC | 24 | Cropland, forest, grassland, shrubland |
| 270 | Slaty-backed Forktail | *Enicurus schistaceus* (Hodgson, 1836) | Eni.sch | LC | LC | 1 | Forest |
| 271 | Little Forktail | *Enicurus scouleri* Vigors, 1832 | Eni.sco | LC | LC | 10 | Cropland, forest, grassland, shrubland |
| 272 | Verditer Flycatcher | *Eumyias thalassinus* Swainson, 1838 | Eum.tha | LC | LC | 36 | Forest, grassland, shrubland, wetland |
| 273 | Red-throated Flycatcher | *Ficedula albicilla* (Pallas, 1811) | Fic.alb | LC | LC | 92 | Cropland, forest, grassland, shrubland, wetland |
| 274 | Slaty-backed Flycatcher | *Ficedula erithacus* (Jerdon and Blyth, 1861) | Fic.eri | LC | NT | 126 | Forest, grassland, shrubland, wetland |
| 275 | Rufous-gorgeted Flycatcher | *Ficedula strophiata* (Hodgson, 1837) | Fic.str | LC | LC | 12 | Forest, grassland, shrubland, wetland |
| 276 | Ultramarine Flycatcher | *Ficedula superciliaris* (Jerdon, 1840) | Fic.sup | LC | LC | 6 | Forest, grassland |
| 277 | Slaty-blue Flycatcher | *Ficedula tricolor* (Hodgson, 1845) | Fic.tri | LC | LC | 13 | Forest, grassland, shrubland |
| 278 | Little Pied Flycatcher | *Ficedula westermanni* (Sharpe, 1888) | Fic.wes | LC | LC | 76 | Cropland, forest, grassland, shrubland, wetland |
| 279 | White-bellied Redstart | *Hodgsonius phaenicuroides* (Gray, 1846) | Hod.pha | LC | LC | 6 | Forest |
| 280 | White-rumped Shama | *Kittacincla malabarica* (Scopoli, 1786) | Kit.mal | LC | LC | 1 | Grassland |
| 281 | Indian Blue Robin | *Larvivora brunnea* Hodgson, 1837 | Lar.bru | LC | LC | 31 | Cropland, forest, grassland, shrubland, wetland |
| 282 | Bluethroat | *Luscinia svecica* (Linnaeus, 1758) | Lus.sve | LC | LC | 9 | Forest, grassland |
| 283 | Blue-capped Rock Thrush | *Monticola cinclorhynchus* (Vigors, 1832) | Mon.cin | LC | LC | 8 | Shrubland, wetland |
| 284 | Chestnut-bellied Rock Thrush | *Monticola rufiventris* (Jardine & Selby, 1833) | Mon.ruf | LC | LC | 11 | Forest, grassland, shrubland |
| 285 | Blue Rock Thrush | *Monticola solitarius* (Linnaeus, 1758) | Mon.sol | LC | LC | 13 | Grassland, shrubland |
| 286 | Asian Brown Flycatcher | *Muscicapa dauurica* Pallas, 1811 | Mus.dau | LC | LC | 18 | Forest, grassland, shrubland, wetland |
| 287 | Ferruginous Flycatcher | *Muscicapa ferruginea* (Hodgson, 1845) | Mus.fer | LC | NT | 33 | Forest, grassland |
| 288 | Dark-sided Flycatcher | *Muscicapa sibirica* Gmelin, 1789 | Mus.sib | LC | LC | 56 | Cropland, forest, grassland, shrubland, wetland |
| 289 | White-tailed Robin | *Myiomela leucura* (Hodgson, 1845) | Myi.leu | LC | LC | 15 | Forest, grassland, shrubland |
| 290 | Blue Whistling Thrush | *Myophonus caeruleus* (Scopoli, 1786) | Myo.cae | LC | LC | 75 | Cropland, forest, grassland, shrubland, wetland |
| 291 | Small Niltava | *Niltava macgrigoriae* (Burton, 1836) | Nil.mac | LC | LC | 11 | Cropland, forest, grassland, shrubland, wetland |
| 292 | Rufous-bellied Niltava | *Niltava sundara* Hodgson, 1837 | Nil.sun | LC | LC | 22 | Forest, grassland, shrubland, wetland |
| 293 | Blue-capped Redstart | *Phoenicurus coeruleocephala* Vigors, 1831 | Pho.coe | LC | LC | 2 | Shrubland |
| 294 | Ferruginous Flycatcher | *Phoenicurus frontalis* Vigors, 1832 | Pho.fro | LC | LC | 8 | Forest, grassland |
| 295 | Plumbeous Water Redstart | *Phoenicurus fuliginosus* (Vigors, 1831) | Pho.ful | LC | LC | 57 | Forest, grassland, shrubland, wetland |
| 296 | Hodgson's Redstart | *Phoenicurus hodgsoni* (Moore, 1854) | Pho.hod | LC | LC | 3 | Forest |
| 297 | White-capped Redstart | *Phoenicurus leucocephalus* (Vigors, 1831) | Pho.leu | LC | LC | 22 | Forest, grassland, shrubland, wetland |
| 298 | Black Redstart | *Phoenicurus ochruros* (Gmelin, 1774) | Pho.och | LC | LC | 78 | Cropland, forest, grassland, shrubland |
| 299 | White-throated Redstart | *Phoenicurus schisticeps* (Gray, 1846) | Pho.sch | LC | LC | 1 | Forest |
| 300 | Pied Bushchat | *Saxicola caprata* (Linnaeus, 1766) | Sax.cap | LC | LC | 62 | Cropland, forest, grassland, shrubland, wetland |
| 301 | Grey Bushchat | *Saxicola ferreus* Gray, 1846 | Sax.fer | LC | LC | 79 | Cropland, forest, grassland, shrubland, wetland |
| 302 | White-tailed Stonechat | *Saxicola leucurus* (Blyth, 1847) | Sax.leu | LC | NT | 30 | Cropland, forest, grassland, wetland |
| 303 | Common Stonechat | *Saxicola torquatus* (Linnaeus, 1766) | Sax.tor | LC | LC | 35 | Cropland, forest, grassland, shrubland, wetland |
| 304 | Golden Bush-robin | *Tarsiger chrysaeus* Hodgson, 1845 | Tar.chr | LC | LC | 2 | Grassland |
| 305 | Orange-flanked Bush-robin | *Tarsiger cyanurus* (Pallas, 1773) | Tar.cya | LC | LC | 1 | Shrubland |
| 306 | White-browed Bush Robin | *Tarsiger indicus* (Vieillot, 1817) | Tar.ind | LC | LC | 44 | Cropland, forest, grassland, shrubland, wetland |
| **Family: Nectariniidae** | | | | | | | |
| 307 | Gould's Sunbird | *Aethopyga gouldiae* (Vigors, 1831) | Aet.gou | LC | LC | 6 | Grassland, forest |
| 308 | Fire-tailed Sunbird | *Aethopyga ignicauda* (Hodgson, 1837) | Aet.ign | LC | LC | 36 | Forest, grassland, shrubland, wetland |
| 309 | Green-tailed Sunbird | *Aethopyga nipalensis* Hodgson, 1837 | Aet.nip | LC | LC | 21 | Forest, cropland, grassland, shrubland |
| 310 | Black-throated Sunbird | *Aethopyga saturata* (Hodgson, 1836) | Aet.sat | LC | LC | 50 | Cropland, forest, grassland, shrubland, wetland |
| 311 | Crimson Sunbird | *Aethopyga siparaja* (Raffles, 1822) | Aet.sip | LC | LC | 27 | Cropland, forest, shrubland |
| 312 | Purple Sunbird | *Cinnyris asiaticus* (Latham, 1790) | Cin.asi | LC | LC | 6 | Forest, shrubland |
| **Family: Oriolidae** | | | | | | | |
| 313 | Indian Golden Oriole | *Oriolus kundoo* Sykes, 1832 | Ori.kun | LC | LC | 54 | Cropland, forest, grassland, shrubland |
| 314 | Eurasian Golden Oriole | *Oriolus oriolus* (Linnaeus, 1758) | Ori.ori | LC | LC | 3 | Forest |
| 315 | Slender-billed Oriole | *Oriolus tenuirostris* Blyth, 1846 | Ori.ten | LC | LC | 45 | Cropland, forest, grassland, shrubland, wetland |
| 316 | Maroon Oriole | *Oriolus traillii* (Vigors, 1832) | Ori.tra | LC | LC | 35 | Forest, shrubland |
| 317 | Black-hooded Oriole | *Oriolus xanthornus* (Linnaeus, 1758) | Ori.xan | LC | LC | 6 | Forest |
| **Family: Paradoxornithidae** | | | | | | | |
| 318 | Great Parrotbill | *Paradoxornis aemodium* Hodgson, 1842 | Con.aem | LC | VU | 14 | Forest, shrubland, wetland |
| 319 | White-browed Fulvetta | *Fulvetta vinipectus* (Hodgson, 1837) | Ful.vin | LC | LC | 10 | Cropland, forest, shrubland |
| 320 | Black-throated Parrotbill | *Paradoxornis nipalensis* (Hodgson, 1837) | Par.nip | LC | LC | 3 | Wetland |
| **Family: Paridae** | | | | | | | |
| 321 | Fire-capped Tit | *Cephalopyrus flammiceps* (Burton, 1836) | Cep.fla | LC | LC | 2 | Shrubland |
| 322 | Gray-crested Tit | *Lophophanes dichrous* (Blyth, 1844) | Lop.dic | LC | LC | 24 | Forest, shrubland, wetland |
| 323 | Black-lored Tit | *Machlolophus xanthogenys* (Vigors, 1831) | Mac.xan | LC | LC | 44 | Cropland, forest, grassland, shrubland, wetland |
| 324 | Great Tit | *Parus major* Linnaeus, 1758 | Par.maj | LC | LC | 160 | Cropland, forest, grassland, shrubland, wetland |
| 325 | Green-backed Tit | *Parus monticolus* Vigors, 1831 | Par.mon | LC | LC | 45 | Forest, grassland, shrubland, wetland |
| 326 | Rufous-vented Tit | *Parus rubidiventris* Blyth, 1847 | Par.rub | LC | LC | 3 | shrubland |
| 327 | Coal Tit | *Periparus ater* (Linnaeus, 1758) | Per.ate | LC | LC | 25 | Forest, shrubland |
| 328 | Rufous-naped Tit | *Periparus rufonuchalis* (Blyth, 1849) | Per.ruf | LC | LC | 4 | Cropland |
| 329 | Yellow-browed Tit | *Sylviparus modestus* E. Burton, 183 | Syl.mod | LC | LC | 3 | Cropland |
| **Family: Passeridae** | | | | | | | |
| 330 | Russet Sparrow | *Passer cinnamomeus* (Temminck, 1836) | Pas.cin | LC | LC | 92 | Cropland, forest, grassland, shrubland, wetland |
| 331 | House Sparrow | *Passer domesticus* (Linnaeus, 1758) | Pas.dom | LC | LC | 165 | Cropland, forest, grassland, shrubland, wetland |
| 332 | Eurasian Tree Sparrow | *Passer montanus* (Linnaeus, 1758) | Pas.mon | LC | LC | 76 | Cropland, forest, grassland, shrubland, wetland |
| **Family: Pellorneidae** | | | | | | | |
| 333 | Puff-throated Babbler | *Pellorneum ruficeps* Swainson, 1832 | Pel.ruf | LC | LC | 33 | Forest, grassland |
| 334 | Rufous-winged Fulvetta | *Schoeniparus castaneceps* (Hodgson, 1837) | Sch.cas | LC | LC | 4 | Cropland, forest |
| **Phylloscopidae** | | | | | | | |
| 335 | Tickell's Leaf Warbler | *Phylloscopus affinis* (Tickell, 1833) | Phy.aff | LC | LC | 60 | Forest, grassland, shrubland, wetland |
| 336 | Green-crowned Warbler | *Phylloscopus burkii* (E. Burton, 1836) | Phy.bur | LC | LC | 14 | Forest |
| 337 | Chestnut-crowned Warbler | *Phylloscopus castaniceps* (Hodgson, 1845) | Phy.cas | LC | LC | 29 | Cropland, forest, grassland, shrubland |
| 338 | Lemon-rumped Warbler | *Phylloscopus chloronotus* (Gray & Gray, 1846) | Phy.chl | LC | LC | 20 | Forest |
| 339 | Common Chiffchaff | *Phylloscopus collybita* (Vieillot, 1817) | Phy.col | LC | LC | 2 | Forest |
| 340 | Dusky Warbler | *Phylloscopus fuscatus* (Blyth, 1842) | Phy.fus | LC | LC | 21 | Forest, grassland, wetland |
| 341 | Hume's Leaf Warbler | *Phylloscopus humei* (Brooks, 1878) | Phy.hum | LC | LC | 25 | Forest, grassland, wetland, shrubland |
| 342 | Ashy-throated Warbler | *Phylloscopus maculipennis* (Blyth, 1867) | Phy.mac | LC | LC | 21 | Forest, grassland, wetland |
| 343 | Large-billed Leaf Warbler | *Phylloscopus magnirostris* Blyth, 1843 | Phy.mag | LC | LC | 8 | Shrubland, grassland |
| 344 | Western Crowned Leaf-warbler | *Phylloscopus occipitalis* (Blyth, 1845) | Phy.occ | LC | LC | 15 | Forest, grassland |
| 345 | Buff-barred Warbler | *Phylloscopus pulcher* Blyth, 1845 | Phy.pul | LC | LC | 5 | Wetland |
| 346 | Blyth's Leaf Warbler | *Phylloscopus reguloides* (Blyth, 1842) | Phy.reg | LC | LC | 53 | Cropland, forest, grassland, shrubland, wetland |
| 347 | Greenish Warbler | *Phylloscopus trochiloides* (Sundevall, 1837) | Phy.tro | LC | LC | 33 | Forest, shrubland |
| 348 | Whistler's Warbler | *Phylloscopus whistleri* (Ticehurst, 1925) | Phy.whi | LC | LC | 14 | Forest |
| 349 | Grey-hooded Warbler | *Phylloscopus xanthoschistos* (Gray, 1846) | Phy.xan | LC | LC | 136 | Cropland, forest, grassland, shrubland, wetland |
| **Family: Pittidae** | | | | | | | |
| 350 | Hooded Pitta | *Pitta sordida* (Müller, 1776) | Pit.sor | LC | VU | 2 | Wetland |
| **Family: Ploceidae** | | | | | | | |
| 351 | Baya Weaver | *Ploceus philippinus* (Linnaeus, 1766) | Plo.phi | LC | NT | 2 | Forest |
| **Family: Pnoepygidae** | | | | | | | |
| 352 | Nepal Cupwing | *Pnoepyga immaculata* Martens & Eck, 1991 | Pno.imm | LC | LC | 2 | Forest |
| **Family: Prunellidae** | | | | | | | |
| 353 | Alpine Accentor | *Prunella collaris* (Scopoli, 1769) | Pru.col | LC | LC | 7 | Wetland, forest |
| 354 | Altai Accentor | *Prunella himalayana* (Blyth, 1842) | Pru.him | LC | LC | 16 | Forest, shrubland |
| **Family: Pycnonotidae** | | | | | | | |
| 355 | Striated Bulbul | *Alcurus striatus* (Blyth, 1842) | Alc.str | LC | LC | 3 | Forest |
| 356 | White-throated Bulbul | *Alophoixus flaveolus* (Gould, 1836) | Alo.fla | LC | LC | 19 | Forest, shrubland |
| 357 | Ashy Bulbul | *Hemixos flavala* Blyth, 1845 | Hem.fla | LC | LC | 33 | Forest, grassland, shrubland |
| 358 | Black Bulbul | *Hypsipetes leucocephalus* (Gmelin, 1789) | Hyp.leu | LC | LC | 61 | Forest, grassland, shrubland, wetland |
| 359 | Mountain Bulbul | *Ixos mcclellandii* (Horsfield, 1840) | Ixo.mcc | LC | LC | 2 | Grassland, shrubland |
| 360 | Red-vented Bulbul | *Pycnonotus cafer* (Linnaeus, 1766) | Pyc.caf | LC | LC | 112 | Cropland, forest, grassland, shrubland, wetland |
| 361 | Red-whiskered Bulbul | *Pycnonotus jocosus* (Linnaeus, 1758) | Pyc.joc | LC | LC | 3 | Forest |
| 362 | Himalayan Bulbul | *Pycnonotus leucogenys* (Gray, 1835) | Pyc.leu | LC | LC | 100 | Cropland, forest, grassland, shrubland, wetland |
| 363 | Black-Crested Bulbul | *Rubigula flaviventris* (Tickell, 1833) | Rub.fla | LC | LC | 9 | Forest |
| **Family: Regulidae** | | | | | | | |
| 364 | Goldcrest | *Regulus regulus* (Linnaeus, 1758) | Reg.reg | LC | LC | 9 | Cropland, forest, shrubland |
| **Family: Rhipiduridae** | | | | | | | |
| 365 | White-throated Fantail | *Rhipidura albicollis* (Vieillot, 1818) | Rhi.alb | LC | LC | 72 | Cropland, forest, grassland, shrubland, wetland |
| **Family: Scotocercidae** | | | | | | | |
| 366 | Black-faced Warbler | *Abroscopus schisticeps* (Gray, 1846) | Abr.sch | LC | LC | 11 | Forest |
| 367 | Grey-sided Bush Warbler | *Cettia brunnifrons* (Hodgson, 1845) | Cet.bru | LC | LC | 12 | Forest, shrubland, cropland, grassland |
| 368 | Chestnut-crowned Bush Warbler | *Cettia major* (F. Moore, 1845) | Cet.maj | LC | LC | 6 | Grassland |
| 369 | Aberrant Bush Warbler | *Horornis flavolivaceus* (Blyth, 1845) | Hor.fla | LC | LC | 49 | Forest, grassland, shrubland, wetland |
| 370 | Chestnut-headed Tesia | *Tesia castaneocoronata* (E. Burton, 1836) | Tes.cas | LC | LC | 2 | Forest |
| 371 | Gray-bellied Tesia | *Tesia cyaniventer* (Hodgson, 1837) | Tes.cya | LC | LC | 20 | Cropland, forest, grassland, shrubland |
| **Family: Sittidae** | | | | | | | |
| 372 | Chestnut-bellied Nuthatch | *Sitta cinnamoventris* Blyth, 1842 | Sit.cin | LC | LC | 4 | Forest |
| 373 | Velvet-fronted Nuthatch | *Sitta frontalis Swainson*, 1820 | Sit.fro | LC | LC | 8 | Forest, shrubland |
| 374 | White-tailed Nuthatch | *Sitta himalayensis* Jardine & Selby, 1835 | Sit.him | LC | LC | 53 | Forest, grassland, shrubland, wetland |
| 375 | Wallcreeper | *Tichodroma muraria* (Linnaeus, 1766) | Tic.mur | LC | LC | 39 | Cropland, forest, shrubland, wetland |
| **Family: Stenostiridae** | | | | | | | |
| 376 | Yellow-bellied Fantail | *Chelidorhynx hypoxanthus* (Blyth, 1843) | Che.hyp | LC | LC | 17 | Forest |
| 377 | Grey-headed Canary Flycatcher | *Culicicapa ceylonensis* (Swainson, 1820) | Cul.cey | LC | LC | 51 | Cropland, forest, shrubland, wetland |
| **Family: Sturnidae** | | | | | | | |
| 378 | Jungle Myna | *Acridotheres fuscus* (Wagler, 1827) | Acr.fus | LC | LC | 75 | Forest, cropland, grassland, shrubland |
| 379 | Bank Myna | *Acridotheres ginginianus* (Latham, 1790) | Acr.gin | LC | LC | 27 | Cropland, forest, grassland, shrubland |
| 380 | Common Myna | *Acridotheres tristis* (Linnaeus, 1766) | Acr.tri | LC | LC | 173 | Cropland, forest, grassland, shrubland, wetland |
| 381 | Asian Pied Starling | *Gracupica contra* (Linnaeus, 1758) | Gra.con | LC | LC | 6 | Shrubland, wetland |
| 382 | Chestnut-tailed Starling | *Sturnia malabarica* (Gmelin, 1789) | Stu.mal | LC | LC | 10 | Forest, grassland |
| 383 | Brahminy Starling | *Sturnus pagodarum* (Gmelin, 1789) | Stu.pag | LC | LC | 41 | Cropland, forest, grassland, shrubland, wetland |
| 384 | Common Starling | *Sturnus vulgaris* Linnaeus, 1758 | Stu.vul | LC | LC | 5 | Forest, wetland |
| **Family: Sylviidae** | | | | | | | |
| 385 | Spotted Bush Warbler | *Bradypterus thoracicus* (Blyth, 1846) | Bra.tho | LC | LC | 15 | Forest, grassland |
| 386 | Lesser Whitethroat | *Curruca curruca* (Linnaeus, 1758) | Cur.cur | LC | LC | 2 | Grassland |
| **Timaliidae** | | | | | | | |
| 387 | Black-chinned Babbler | *Cyanoderma pyrrhops* (Blyth, 1844) | Cya.pyr | LC | LC | 7 | Forest, shrubland |
| 388 | Pin-striped Tit Babbler | *Mixornis gularis* (Horsfield, 1822) | Mix.gul | LC | LC | 4 | Shrubland |
| 389 | Rusty-cheeked Scimitar Babbler | *Pomatorhinus erythrogenys* Vigors, 1832 | Pom.ery | LC | LC | 6 | Forest |
| 390 | Streak-breasted Scimitar Babbler | *Pomatorhinus ruficollis* Hodgson, 1836 | Pom.ruf | LC | LC | 4 | Forest |
| 391 | White-browed Scimitar- babbler | *Pomatorhinus schisticeps* Hodgson, 1836 | Pom.sch | LC | LC | 2 | Forest |
| 392 | Gray-throated Babbler | *Stachyris nigriceps* Blyth, 1844 | Sta.nig | LC | LC | 18 | Cropland, forest, shrubland |
| 393 | Chestnut-capped Babbler | *Timalia pileata* Horsfield, 1821 | Tim.pil | LC | NT | 5 | Forest |
| **Family: Troglodytidae** | | | | | | | |
| 394 | Winter Wren | *Troglodytes hiemalis* Vieillot, 1819 | Tro.hie | LC | LC | 3 | Shrubland |
| **Family: Turdidae** | | | | | | | |
| 395 | Orange-headed Thrush | *Geokichla citrina* (Latham, 1790) | Geo.cit | LC | LC | 51 | Cropland, forest, grassland, shrubland |
| 396 | Pied Thrush | *Geokichla wardii* (Blyth, 1842) | Geo.war | LC | LC | 15 | Forest, grassland |
| 397 | White-collared Blackbird | *Turdus albocinctus* Royle, 1840 | Tur.alb | LC | LC | 46 | Cropland, forest, grassland, shrubland |
| 398 | Black-throated Thrush | *Turdus atrogularis* Jarocki, 1819 | Tur.atr | LC | LC | 23 | Forest, grassland, shrubland, wetland |
| 399 | Gray-winged Blackbird | *Turdus boulboul* (Latham, 1790) | Tur.bou | LC | LC | 24 | Cropland, forest, grassland, shrubland |
| 400 | Dusky Thrush | *Turdus eunomus* Temminck, 1831 | Tur.eun | LC | LC | 5 | Forest, grassland, shrubland |
| 401 | Eurasian Blackbird | *Turdus merula* Linnaeus, 1758 | Tur.mer | LC | LC | 23 | Forest, grassland, shrubland |
| 402 | Red-throated Thrush | *Turdus ruficollis* Pallas, 1776 | Tur.ruf | LC | LC | 3 | Wetland |
| 403 | Mistle Thrush | *Turdus viscivorus* Linnaeus, 1758 | Tur.vis | LC | LC | 2 | Shrubland |
| 404 | Scaly Thrush | *Zoothera dauma* (Latham, 1790) | Zoo.dau | LC | LC | 24 | Forest, grassland, shrubland, wetland |
| 405 | Dark-sided Thrush | *Zoothera marginata* Blyth, 1847 | Zoo.mar | LC | VU | 28 | Forest, grassland, shrubland, wetland |
| 406 | Alpine Thrush | *Zoothera mollissima* (Blyth, 1842) | Zoo.mol | LC | LC | 3 | Shrubland |
| 407 | Long-billed Thrush | *Zoothera monticola* Vigors, 1832 | Zoo.mon | LC | LC | 8 | Cropland, grassland |
| **Family: Vangidae** | | | | | | | |
| 408 | Large Woodshrike | *Tephrodornis virgatus* (Temminck, 1824) | Tep.vir | LC | LC | 2 | Forest |
| **Family: Vireonidae** | | | | | | | |
| 409 | White-bellied Erpornis | *Erpornis zantholeuca* (Blyth, 1844) | Erp.zan | LC | LC | 3 | Forest |
| 410 | White-bellied Erpornis | *Yuhina zantholeuca* (Blyth, 1844) | Yuh.zan | LC | LC | 13 | Forest, shrubland |
| **Family: Zosteropidae** | | | | | | | |
| 411 | Whiskered Yuhina | *Yuhina flavicollis* Hodgson, 1836 | Yuh.fla | LC | LC | 39 | Forest, shrubland, wetland |
| 412 | Stripe-throated Yuhina | *Yuhina gularis* Hodgson, 1836 | Yuh.gul | LC | LC | 2 | shrubland |
| 413 | Rufous- vented Yuhina | *Yuhina occipitalis* Hodgson, 1836 | Yuh.occ | LC | LC | 3 | Shrubland |
| 414 | Oriental White-eye | *Zosterops palpebrosus* (Temminck, 1824) | Zos.pal | LC | LC | 90 | Cropland, forest, grassland, shrubland, wetland |
| **Order: Pelecaniformes** | | | | | | | |
| **Family: Ardeidae** | | | | | | | |
| 415 | Gray Heron | *Ardea cinerea* Linnaeus, 1758 | Ard.cin | LC | LC | 19 | Wetland |
| 416 | Purple Heron | *Ardea purpurea* Linnaeus, 1766 | Ard.pur | LC | LC | 2 | Wetland |
| 417 | Green Backed Heron | *Butorides striata* (Linnaeus, 1758) | But.str | LC | LC | 1 | Forest |
| 418 | Cinnamon Bittern | *Ixobrychus cinnamomeus* (Gmelin, 1789) | Ixo.cin | LC | LC | 2 | Shrubland |
| 419 | Black-crowned Night Heron | *Nycticorax nycticorax* (Linnaeus, 1758) | Nyc.nyc | LC | LC | 2 | Forest |
| **Order: Piciformes** | | | | | | | |
| **Family: Megalaimidae** | | | | | | | |
| 420 | Golden-throated Barbet | *Megalaima franklinii* (Blyth, 1842) | Meg.fra | LC | LC | 10 | Forest, shrubland |
| 421 | Blue-throated Barbet | *Psilopogon asiaticus* (Latham, 1790) | Psi.asi | LC | LC | 26 | Cropland, forest, grassland, shrubland, wetland |
| 422 | Coppersmith Barbet | *Psilopogon haemacephalus* (Müller, 1776) | Psi.hae | LC | LC | 34 | Forest, grassland,shrubland, wetland |
| 423 | Lineated Barbet | *Psilopogon lineatus* (Vieillot, 1816) | Psi.lin | LC | LC | 21 | Cropland, forest, grassland, wetland |
| 424 | Great Barbet | *Psilopogon virens* (Boddaert, 1783) | Psi.vir | LC | LC | 41 | Cropland, forest, grassland, shrubland, wetland |
| **Family: Picidae** | | | | | | | |
| 425 | Bay Woodpecker | *Blythipicus pyrrhotis* (Hodgson, 1837) | Bly.pyr | LC | NT | 6 | Forest, shrubland |
| 426 | Greater Flameback | *Chrysocolaptes guttacristatus* (Tickell, 1833) | Chr.gut | LC | LC | 11 | Grassland, forest, shrubland |
| 427 | Greater Yellownape | *Chrysophlegma flavinucha* (Gould, 1834) | Chr.fla | LC | LC | 50 | Cropland, forest, shrubland, wetland |
| 428 | Gray-capped Pygmy Woodpecker | *Dendrocopos canicapillus* (Blyth, 1845) | Den.can | LC | LC | 5 | Forest |
| 429 | Darjeeling Woodpecker | *Dendrocopos darjellensis* (Blyth, 1845) | Den.dar | LC | LC | 8 | Shrubland |
| 430 | Fulvous-breasted Woodpecker | *Dendrocopos macei* (Vieillot, 1818) | Den.mac | LC | LC | 42 | Cropland, forest, grassland, shrubland, wetland |
| 431 | Gray-headed Woodpecker | *Dendropicos spodocephalus* (Bonaparte, 1850) | Den.spo | LC | LC | 49 | Cropland, forest, grassland, shrubland |
| 432 | Black-rumped Flameback | *Dinopium benghalense* (Linnaeus, 1758) | Din.ben | LC | LC | 7 | Cropland, forest, grassland, shrubland, wetland |
| 433 | Himalayan Flameback | *Dinopium shorii* (Vigors, 1832) | Din.sho | LC | LC | 1 | Cropland |
| 434 | Crimson-breasted Woodpecker | *Dryobates pernyii* (Verreaux, 1867) | Dry.per | LC | LC | 2 | Forest, wetland |
| 435 | Eurasian Wryneck | *Jynx torquilla* Linnaeus, 1758 | Jyn.tor | LC | LC | 2 | Forest |
| 436 | Brown-fronted Woodpecker | *Leiopicus auriceps* (Vigors, 1831) | Lei.aur | LC | LC | 25 | Cropland, forest, grassland, shrubland |
| 437 | Rufous Woodpecker | *Micropternus brachyurus* (Vieillot, 1818) | Mic.bra | LC | LC | 41 | Cropland, forest, grassland, shrubland |
| 438 | Lesser Yellownape | *Picus chlorolophus* Vieillot, 1818 | Pic.chl | LC | LC | 12 | Forest, grassland, shrubland, wetland |
| 439 | Scaly-bellied Woodpecker | *Picus squamatus* Vigors, 1831 | Pic.squ | LC | LC | 3 | Shrubland, grassland |
| 440 | Streak-throated Woodpecker | *Picus xanthopygaeus* (Gray & Gray, 1846) | Pic.xan | LC | LC | 2 | Wetland |
| **Order: Podicipediformes** | | | | | | | |
| **Family: Podicipedidae** | | | | | | | |
| 441 | Great Crested Grebe | *Podiceps cristatus* (Linnaeus), 1758 | Pod.cri | LC | LC | 21 | Wetland |
| 442 | Little Grebe | *Tachybaptus ruficollis* (Pallas, 1764) | Tac.ruf | LC | LC | 33 | Wetland |
| **Order: Psittaciformes** | | | | | | | |
| **Family: Psittacidae** | | | | | | | |
| 443 | Rose-ringed Parakeet | *Alexandrinus krameri* (Scopoli, 1769) | Ale.kra | LC | LC | 69 | Cropland, forest |
| 444 | Plum-headed Parakeet | *Himalayapsitta cyanocephala* (Linnaeus, 1766) | Him.cya | LC | LC | 72 | Cropland, forest, grassland, shrubland, wetland |
| 445 | Slaty-headed Parakeet | *Himalayapsitta himalayana* (Lesson, 1832) | Him.him | LC | LC | 87 | Cropland, forest, grassland, shrubland, wetland |
| 446 | Brown Parrotbill | *Poicephalus meyeri* (Cretzschmar, 1827) | Poi.mey | LC | VU | 2 | Shrubland |
| 447 | Red-breasted Parakeet | *Psittacula alexandri* (Linnaeus, 1758) | Psi.ale | NT | VU | 165 | Cropland, forest, grassland |
| **Order: Strigiformes** | | | | | | | |
| **Family: Strigidae** | | | | | | | |
| 448 | Spotted Owlet | *Athene brama* (Temminck, 1821) | Ath.bra | LC | LC | 18 | Cropland, forest, grassland, shrubland, wetland |
| 449 | Collared Owlet | *Glaucidium brodiei* (Burton, 1836) | Gla.bro | LC | LC | 105 | Cropland, forest, grassland, shrubland, wetland |
| 450 | Asian Barred Owlet | *Glaucidium cuculoides* (Vigors, 1831) | Gla.cuc | LC | LC | 46 | Cropland, forest, grassland, shrubland, wetland |
| 451 | Jungle Owlet | *Glaucidium radiatum* (Tickell, 1833) | Gla.rad | LC | LC | 4 | Cropland, forest |
| 452 | Brown Fish Owl | *Ketupa zeylonensis* (Gmelin, 1788) | Ket.zey | LC | VU | 1 | Forest |
| 453 | Brown Boobook | *Ninox scutulata* (Raffles, 1822) | Nin.scu | LC | LC | 8 | Forest, shrubland |
| 454 | Collared Scops-owl | *Otus lettia* (Hodgson, 1836) | Otu.let | LC | LC | 2 | Shrubland |
| **Order: Suliformes** | | | | | | | |
| **Family: Anhingidae** | | | | | | | |
| 455 | Oriental Darter | *Anhinga melanogaster* Pennant, 1769 | Anh.mel | NT | NT | 4 | Forest, grassland |
| **Family: Phalacrocoracidae** | | | | | | | |
| 456 | Great Cormorant | *Phalacrocorax carbo* (Linnaeus, 1758) | Pha.car | LC | NT | 125 | Wetland, forest, grassland |
| 457 | Little Cormorant | *Phalacrocorax niger* (Gmelin 1789) | Pha.nig | LC | LC | 23 | Wetland |
| **Order: Trogoniformes** | | | | | | | |
| **Family: Trogonidae** | | | | | | | |
| 458 | Red-headed Trogon | *Harpactes erythrocephalus* (Gould, 1834) | Har.ery | LC | EN | 1 | Forest |
